# Puma habitat preferences when moving and feeding predict the potential for human–carnivore conflict in shared landscapes

**DOI:** 10.1002/eap.70101

**Published:** 2025-09-11

**Authors:** Justin P. Suraci, L. Mae Lacey, Patrick T. Freeman, Andrew Stratton, Caitlin Kupar, Kimberly Sager‐Fradkin, Dylan Bergman, Bethany Ackerman, Kristen A. Phillips, Shannon Murphie, Cassandra Sullivan, L. Mark Elbroch

**Affiliations:** ^1^ Conservation Science Partners, Inc. Truckee California USA; ^2^ Panthera New York New York USA; ^3^ Lower Elwha Klallam Tribe Port Angeles Washington USA; ^4^ Point No Point Treaty Council Poulsbo Washington USA; ^5^ Skokomish Indian Tribe Skokomish Washington USA; ^6^ Quinault Division of Natural Resources Taholah Washington USA; ^7^ Makah Tribe Neah Bay Washington USA; ^8^ Port Gamble S'Klallam Tribe Kingston Washington USA

**Keywords:** behavior‐specific habitat selection, carnivore ecology, human–wildlife coexistence, integrated step selection analysis, resources selection function

## Abstract

Large carnivore persistence in an increasingly human‐dominated world requires coexistence between carnivores and people on shared landscapes. Yet, sharing space with carnivores presents challenges, including maintaining sufficient habitat to allow carnivores to satisfy life‐history needs (e.g., hunting, dispersal, and territory establishment) while avoiding conflict with people. To understand the drivers of carnivore habitat use and conflict in shared landscapes, we quantified puma (*Puma concolor*) habitat selection while moving and while feeding on native prey across a mosaic of developed areas, working landscapes, and wildlands on the Olympic Peninsula, Washington, USA. We fit resource selection models to movement data from GPS collars and to kill site locations for pumas across four age‐sex classes: male and female adults and dispersers. We then quantified the association between habitat preferences for each behavioral state (moving and feeding) and the spatial distribution of puma–human interactions reported to state wildlife authorities. Across age‐sex classes, puma habitat selection was more strongly influenced by human land uses when moving than when feeding, with adult females being the only age‐sex class to exhibit avoidance of development and agriculture when feeding. Correspondingly, areas categorized as highly suitable for feeding but unsuitable for movement tended to have substantially greater amounts of developed and agricultural land than areas considered suitable for both behaviors. Analysis of puma–human interactions revealed that habitat preferences when feeding were strongly associated with the probability of both domestic animal depredations and sightings of pumas by people across most puma age‐sex classes (except adult females). By contrast, habitat selection when moving was negatively associated with depredations and sightings for all pumas. These findings suggest that pumas are encountering livestock, pets, and people opportunistically in areas that are otherwise highly suitable for hunting native prey, but that sensitivity to human disturbance when moving across the landscape leads to limited opportunity for conflict when engaged in this behavior. We leveraged these findings to identify important multifunctional habitat across our study area (i.e., places that will support both moving and feeding) and to explore pathways toward stable puma–human coexistence based on achievable changes to human behavior that minimize conflict opportunities.

## INTRODUCTION

Coexistence with humans on shared landscapes is critical to the persistence of large carnivores (Chapron & López‐Bao, 2016; Lamb et al., 2020) and also provides ecological and societal benefits. Carnivores play a crucial role in influencing ecosystem dynamics (Ripple et al., 2014), and their impacts on prey, particularly ungulates, may provide important services to human communities by reducing wildlife damage (e.g., crop raiding or vehicle collisions; Gilbert et al., 2017, 2021). However, coexistence with large carnivores is often controversial due to the potential for conflict with humans, including predation on livestock (König et al., 2020; Wilkinson et al., 2020) and real or perceived threats to human safety (Bombieri et al., 2018). Indeed, human–carnivore conflict and resulting lethal control of the animals involved is a primary source of mortality for many carnivore populations (Benson et al., 2023; Elbroch & Treves, 2023). The rapid expansion of development into wildland areas (Burke et al., 2021) means that wide‐ranging carnivore species are increasingly reliant on a mosaic of natural and modified landscapes in close proximity to human communities (Lamb et al., 2020; Suraci et al., 2020), highlighting the urgency of identifying strategies that mitigate human–carnivore conflict and promote coexistence (Carter & Linnell, 2023).

Fundamental to building such strategies is understanding habitat preferences of carnivores living in human‐dominated landscapes, which can provide key insights into where and when human–carnivore conflict is most likely to occur (Maletzke et al., 2017; Wilkinson et al., 2020). Large carnivores will often avoid areas of high human activity (Gehr et al., 2017; Nisi, Suraci, et al., 2022); however, the degree to which carnivores avoid (or are attracted to) human disturbance may depend on the behavior in which the animal is engaged (Abrahms et al., 2016; Suraci, Frank, et al., 2019). When traveling during territorial patrols or transiting between feeding and resting sites, carnivores may accept higher movement costs to reduce the risk of encountering humans. For example, they may use more energetically demanding but less developed movement routes (e.g., steeper slopes, more rugged terrain; Nisi, Suraci, et al., 2022) or adjust their movement patterns (e.g., speed, tortuosity) to increase crypsis and minimize time in areas of high human activity (Gehr et al., 2017; Nickel et al., 2021).

By contrast, carnivores may be attracted to human‐dominated landscapes because of high prey availability or favorable hunting conditions (e.g., edge habitats) (Buderman et al., 2018; Ditmer et al., 2021; Gehr et al., 2017; Kertson et al., 2011), particularly as hunger levels increase (Blecha et al., 2018). For instance, African lions (*Panthera leo*) tended to avoid human activity associated with livestock herding when traveling or resting but selected for these areas when feeding at night (Suraci, Frank, et al., 2019). Both Eurasian lynx (*Lynx lynx*) and wolves (*Canis lupus*) have been shown to reduce avoidance of, or even select for, areas of elevated human risk when making kills, particularly when the availability of primary prey (i.e., wild ungulates) in these areas is high relative to less disturbed habitats (Barker et al., 2023; Gehr et al., 2017).

However, it remains unclear the degree to which habitat preferences during movement versus when hunting wild prey are associated with human–carnivore conflict. For some carnivore populations, predation on alternative prey, including domestic animals, may be largely opportunistic (Alldredge et al., 2019; Cristescu et al., 2019) and could therefore occur outside of preferred hunting habitat. Direct encounters between carnivores and people could conceivably occur at high rates across the spectrum of human development levels, either being highest in close proximity to developed areas where human activity is concentrated (Alldredge et al., 2019), in intermediate zones with low housing density (Kertson et al., 2011; Maletzke et al., 2017), or in more remote recreation areas where carnivores are known to exploit trails and roads for movement (Nickel et al., 2020; Sweanor et al., 2008). Quantifying behavior‐specific habitat preferences may therefore be an important first step toward understanding spatial variation in the likelihood of human–carnivore interactions and the potential for conflict.

Behavioral differences between carnivore sexes and life‐history stages may be another key factor determining both habitat requirements and the potential for conflict (Alldredge et al., 2019; Elbroch & Treves, 2023; Kertson et al., 2013; Peebles et al., 2013). As they traverse unknown areas and attempt to avoid dominant conspecifics, dispersing individuals will frequently utilize lower quality, riskier, and/or more disturbed habitats relative to those selected by settled adults (Abrahms et al., 2017; Elliot et al., 2014). Prey choice by younger, inexperienced carnivores may also differ from that of adults, with younger individuals potentially engaging riskier prey (Elbroch et al., 2017) and interacting more frequently with domestic animals (Peebles et al., 2013). Habitat use and potential overlap with humans may also differ between sexes, for instance, with male carnivores potentially using larger areas than females as they defend breeding territories or seek out mates. These increased space requirements may lead to reduced avoidance of anthropogenic landscapes by males (Elliot et al., 2014; Suraci et al., 2020). However, the association between sex‐specific habitat use and conflict potential remains relatively unexplored (Alldredge et al., 2019; Teichman et al., 2013) and likely differs both among and within carnivore populations (Berezowska‐Cnota et al., 2023).

Pumas (*Puma concolor*) have the largest geographic range of any carnivore species in the western hemisphere and often occur in or near modified and working landscapes (LaBarge et al., 2022). This species is therefore frequently implicated in human–wildlife conflict, particularly livestock predation (Guerisoli et al., 2021) as well as potentially dangerous encounters with humans (Mattson et al., 2011; Wang et al., 2019). For pumas living in proximity to people, conflict‐related retaliatory killings are often a primary cause of death (Dellinger et al., 2021; LaBarge et al., 2022; Nisi, Benson, & Wilmers, 2022). In California, USA, where puma hunting is illegal, the intentional killing of pumas following livestock predation was the most frequently reported source of mortality (Benson et al., 2023). Human‐caused mortality not only affects puma abundance, survival, and population viability (Benson et al., 2023; Erwin et al., 2024; Nisi et al., 2023), but can substantially impact individual behavior and habitat use. Pumas exhibit fear responses to humans (Suraci, Clinchy, et al., 2019) that impact hunting and feeding behavior (Smith et al., 2015, 2017), habitat selection (Nisi, Suraci, et al., 2022), and large‐scale space use patterns (e.g., home range size; Nickel et al., 2021). Yet, pumas increasingly overlap with humans across western North America as human populations increase, available habitat decreases, and the species recolonizes portions of its historical range (LaBarge et al., 2022). Pumas (particularly dispersing individuals) may in some cases benefit from utilizing modified environments through access to alternative prey or beneficial hunting conditions such as habitat edges (Alldredge et al., 2019; Blecha et al., 2018; Ditmer et al., 2021; Kertson et al., 2011).

Here, we examined puma habitat selection and potential for conflict while moving and feeding on kills across a mosaic of developed areas, working landscapes, and wildlands on the Olympic Peninsula in Washington, USA. We quantified selection for multiple age‐sex classes (female and male adults and dispersers) that may differ in habitat requirements and conflict potential. We then associated the probability of selection while moving or feeding with the spatial distribution of puma‐related incidents reported to the state authorities. We focused on two categories of reported incidents: (1) attacks on livestock or pets and (2) puma sightings. While the latter does not necessarily imply conflict, the incidence of sightings may relate to the risks perceived by local residents from living with carnivores, which can shape social attitudes toward coexisting with predators (Bombieri et al., 2018; Treves & Bruskotter, 2014). We hypothesized that (1) pumas would generally exhibit stronger avoidance of anthropogenic landscape features when moving relative to feeding, given the potential for increased prey availability or hunting success in modified habitats (Ditmer et al., 2021; Kertson et al., 2011), and that (2) dispersing animals would be more likely than adults to use risky areas near people given their lack of familiarity with the landscape and/or generally lower risk aversion (Elbroch & Treves, 2023; Peebles et al., 2013). Given Hypothesis 1 above, we further hypothesized that (3) incidents of conflict with domestic animals would be more likely to occur where habitat suitability for puma feeding is high (Alldredge et al., 2019; Cristescu et al., 2019), but that (4) habitat suitability for moving would be more strongly associated with reported puma sightings as pumas overlap with recreationists in wildland areas and along habitat edges (Maletzke et al., 2017).

## METHODS

### Puma movement and kill site datasets

**FIGURE 1:**
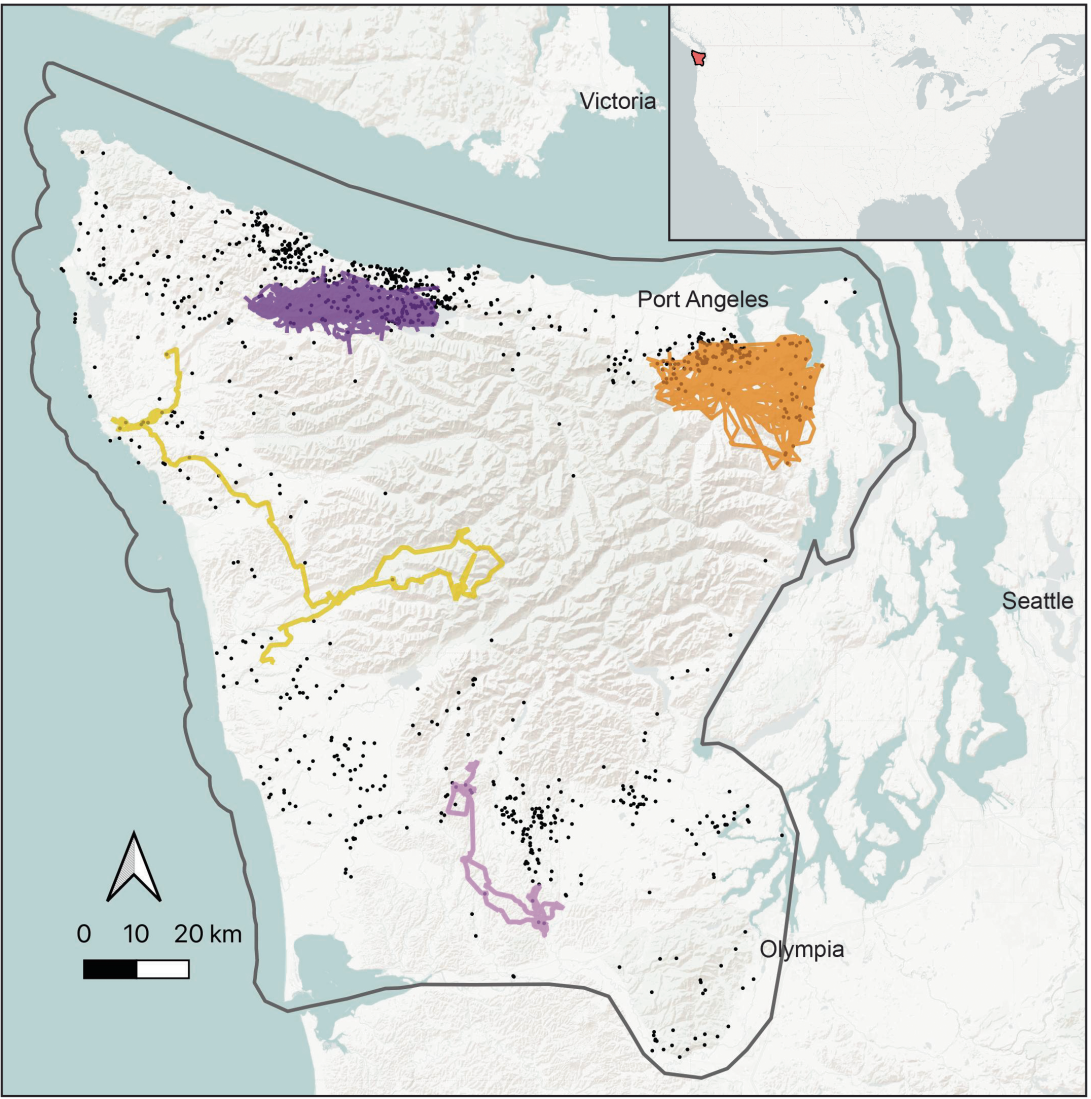
Map of the study area on the Olympic Peninsula, Washington, USA. Movement tracks are shown for four example pumas, an adult female (dark purple), adult male (orange), dispersing female (light purple), and dispersing male (yellow). Points illustrate the locations of all puma kill sites included in the analysis. The inset shows the location of the study area in North America.

We captured pumas on the Olympic Peninsula of Washington, USA (Figure 1) using trailing hounds (see Appendix S1, Section S1: Supplementary Methods for capture details) and fitted them with Lotek LiteTrack 420 or Vectronics Vertex Iridium collars programmed to gather data at either one‐hour or two‐hour intervals. We performed data cleaning on an initial GPS collar dataset from 93 individual pumas collared between January 1, 2017 and September 29, 2022, and segmented each puma movement track into those points associated with two life stages—dispersal and adulthood—using field records and a net squared displacement‐based modeling approach, as described in detail in Appendix S1, Section S1: Supplementary Methods. Our final dataset included 63 unique individuals, nine of which were collared during both disperser and adult life stages. Final sample sizes for all age and sex classes were: adult female, *n* = 26 individuals; adult male, *n* = 19; dispersing female, *n* = 9; dispersing male, *n* = 18.

To identify locations where pumas spend time feeding on prey, we identified putative kill sites from puma GPS data clusters using the GPSeqClus package (Clapp et al., 2021) in R. Putative kill sites were then investigated by field teams on the ground to confirm the presence of a kill and prey species. Analyses focused on kills made by the 63 pumas in the final collar dataset between April 10, 2018 and October 22, 2022, resulting in *n* = 1103 investigated kill sites (adult female, *n* = 504; adult male, *n* = 309; disperser female, *n* = 107; and disperser male, *n* = 183). The large majority (97.7%) of investigated kills were of wild prey, primarily ungulates (black‐tailed deer *Odocoileus hemionus columbianus* and Roosevelt elk *Cervus canadensis roosevelti*; *n* = 882 kills [80.0% of total]), with only 1.5% of kills being recorded as domestic prey (domestic cats or livestock, *n* = 17). These domestic prey kills were retained in our kill site dataset given their very low proportional representation and, as described below, we confirmed that their retention had a negligible influence on our results.

### Habitat covariates

We developed a set of covariates that represented natural landscape features (i.e., “nonhuman” covariates) as well as anthropogenic influences (“human” covariates) across the Olympic Peninsula during our study period (2017–2022). Nonhuman covariates included annually derived tree cover and forest edge density; shrub percent cover; terrain slope, ruggedness and topographic position index (TPI); and distance to riparian areas. Human covariates included development and agricultural cover, distance to development and agriculture, distance to nearest major road (i.e., Washington state routes), and a “forestry activity” layer, representing locations within our study area experiencing active forestry during the study period (2018–2022). All covariates are described in detail in Appendix S1, Section S1: Supplementary Methods and Table S1. Covariate preparation was conducted in Google Earth Engine (Gorelick et al., 2017), and final covariate layers were projected to “NAD83 UTM Zone 10N” and exported at 30‐m resolution.

### Movement‐based habitat selection

We performed integrated step selection analyses (iSSA; Avgar et al., 2016) with puma GPS location data. iSSA compares used steps (straight line between subsequent GPS locations) to available steps that the puma could have taken from the same starting location. The probability a step is used is modeled as a function of environmental covariates and movement parameters (step length, turning angle), thus capturing resource selection and selection‐independent drivers of animal movement (Avgar et al., 2016). We developed five sets of iSSA models, first fitting models to data pooled across all age and sex classes (pooled models) and then to data for each of the four age‐sex classes separately (adult females, adult males, dispersing females, and dispersing males). Prior to model fitting, we removed all two‐hour locations with step lengths <20 m in an effort to exclude stationary points that do not reflect independent movement decisions. This 20‐m cut‐off has been used in prior analyses on pumas (Nisi, Suraci, et al., 2022) and other large felids (Suraci, Frank, et al., 2019) to differentiate stationary and movement points. We simulated 10 random available steps for each used step, with step lengths and turning angles drawn from a gamma distribution and a von Mises distribution, respectively, with distributions parameterized using empirical step length and turning angle values (Avgar et al., 2016; Signer et al., 2019).

For each used and available step, terrain variable values (slope, ruggedness, and TPI) were extracted from each pixel along the path and averaged to generate the covariate value for a given step. All distance covariates (e.g., distance to developed areas) were calculated from the end point of each used and available step. We attempted to account for the scale at which pumas respond most strongly to a given habitat covariate (i.e., the “scale of effect”; Jackson & Fahrig, 2015) by calculating all percent cover variables (Appendix S1: Table S1) at two scales—150 m and 1 km—capturing the influence of the immediate environment and the broader landscape, respectively. To identify the scale of effect for each habitat covariate, we fit single variable models (using the modeling approach described in detail below), modeling the probability that each step was used (1) or available (0) as a function of a single habitat covariate at one of the two scales. We then compared the two single‐variable models for a given habitat covariate using Akaike's Information Criterion corrected for small sample size (AIC_c_; Burnham & Anderson, 2002), and considered the model with the lowest AIC_c_ score to represent the most influential scale for the habitat covariate in question, which was then used in subsequent modeling stages.

Following Muff et al. (2020), we fit iSSA models using mixed effects Poisson regression, the likelihood equivalent of the conditional logistic regression approach traditionally used in step selection designs (Fortin et al., 2005). We included individual‐level random slopes for each habitat covariate to account for nonindependence between data points collected from the same animal, and fit random intercepts for each “stratum,” that is, the associated set of one used and ten available steps. We fixed random intercept variance at a high value (1000) to avoid shrinkage toward zero (Muff et al., 2020). For pooled data and for each age‐sex class, we fit multiple models with various combinations of human and nonhuman habitat covariates (see two‐stage model selection approach described below), and included the log of step length and the cosine of turning angle in each model to account for movement parameters (Avgar et al., 2016). We prepared datasets for iSSA modeling using the *amt* package in R (Signer et al., 2019) and fit models using the *glmmTMB* package (Brooks et al., 2023).

### Feeding site selection

Verified kill locations represent places where pumas spent time feeding on prey and were used here to quantify habitat selection while feeding. Predation events represent the outcome of multiple linked processes, including spatial overlap between predator and prey, direct encounter, and successful attack (Suraci et al., 2022). Here we assume that the spatial distribution of kill sites reflects puma attempts to increase the likelihood of each process by selecting for (1) habitat features associated with high prey availability (thus increasing the likelihood of overlap and encounter) and (2) habitat where hunting success is high, given that prey are available, while also accounting for other considerations that affect feeding site quality (e.g., risk from humans). We used resource selection function (RSF) models (Fieberg et al., 2021; Manly et al., 2002) to compare used kill locations to a set of available locations. We derived available locations by first fitting minimum convex polygons (MCPs) around all GPS locations for a given puma and randomly sampling 10 available locations within that MCP for each kill made by that puma. This process was performed separately for each life stage when the same individual was collared as a disperser and an adult. As above, we developed five sets of RSF models using pooled data (all age‐sex classes) and data for each age‐sex class individually. Habitat covariates were extracted at each used and available point, and we again accounted for the scale of effect by summarizing all percent cover covariates within 100 m, 500 m, and 1 km of each point and using the process described above to identify the appropriate scale for each covariate. We fit mixed effects logistic regression models to used (1) and available (0) points using the *blme* package in R (Dorie et al., 2021), including individual‐level random slopes for each covariate (Muff et al., 2020). We again fit multiple RSF models for each pooled and age‐sex class‐specific dataset, as described in the next section. As noted above, our kill site dataset included a very small number of domestic animal kill sites (*n* = 17, 1.5% of all kills). To confirm that these few kills did not affect our overall results, we re‐ran the full model selection process for the pooled dataset after removing these 17 domestic kills, as well as nine kills classed as “unknown” (26 kills total, 2.3% of the dataset).

### Model selection, validation, and inference

For each dataset (pooled, age‐sex class‐specific) and model type (movement iSSA, feeding site RSF), we employed a two‐stage modeling approach, first fitting separate sets of models to identify the top set of human and nonhuman covariates (Stage 1). We then combined the covariates from these top models into a set of “combination” models (Stage 2), which were compared using AIC_c_ to arrive at the final model for each dataset. The model sets considered in each stage are presented in Appendix S1: Tables S2–S5. We then validated the final model for each dataset using k‐fold cross‐validation to estimate model predictive performance. Model selection and validation details are given in Appendix S1, Section S1: Supplementary Methods. All results presented below are based on the final validated models for each dataset.

We used each final model to map relative probability of selection, *w*(***x***) (a proxy for relative habitat suitability for movement or feeding), across the study area based on the set of habitat covariates, ***x***, available in each 30‐m pixel. Following Manly et al. (2002) the relative probability of selection at a given location, *i*, is given by.

(1) $$w\left(x_{i}\right)=\exp\left(\beta_{1}x_{1i}+\cdots +\beta_{n}x_{\mathrm{ni}}\right),$$

where βs are the parameter estimates from the top model for a given dataset. While predicting from Equation (1) is standard practice for generating maps of habitat suitability from RSF‐type models, predicting from iSSAs presents additional challenges. Unlike with an RSF, the distribution of available locations in a step selection analysis is nonstatic and dependent on an animal's current location (Fieberg et al., 2021; Florko et al., 2024). While simulation approaches exist for deriving individual‐level utilization distributions (UDs) from fitted iSSA models (Signer et al., 2017, 2024), we were primarily interested in using predictions from the population‐level iSSA as a proxy for where pumas are likely to engage in movement behavior, rather than developing individual UDs. We have therefore chosen to move forward with predicting from Equation (1) based on our top fitted iSSA models, but have taken two additional steps to ensure that the inferences from these prediction surfaces are reasonable. First, we have performed an additional out‐of‐sample validation procedure to assess the ability of our iSSA prediction surfaces to capture movement habitat suitability for individuals not included in the model. We fit the top pooled iSSA model using data from a subset of individuals (*n* = 50 pumas), derived the habitat suitability surface via Equation (1), and quantified predicted habitat suitability at locations used by the remaining, out‐of‐sample individuals (*n* = 13 pumas). This validation procedure is described in detail in Appendix S1: Section S3 and confirmed that the prediction surface performs well, with used locations from out‐of‐sample individuals tending to fall in areas with the highest predicted values of habitat suitability (see Appendix S1: Table S3). Second, given that the outputs of RSF‐ and iSSA‐type models are not interchangeable (due to the differences in used and available distributions noted above), we rescaled predicted habitat suitability values from each model type to a common scale (i.e., units of one standard deviation) prior to incorporating them into downstream analyses and consider these to be estimates of the relative suitability of habitat for a given behavior type.

Using the prediction surfaces generated via Equation (1), we compared spatial patterns of habitat selection when moving versus feeding at kills by reclassifying the predicted *w*(***x***) value for a given model into three categories—low, moderate, and high—based on 33.3% and 66.6% quantiles calculated across all pixels in the study area. We then overlaid the reclassified outputs for each paired set of movement‐based and feeding site models (e.g., pooled iSSA prediction overlaid with pooled RSF prediction, adult female iSSA overlaid with adult female RSF) and identified locations across the study area where *w*(***x***) was high for both movement and feeding site selection or where *w*(***x***) was high for one and low for the other (i.e., high for movement selection and low for feeding site selection or vice versa). For the pooled models, we then related these spatial patterns in high versus low relative values of *w*(***x***) back to environmental covariates by randomly sampling 1000 points within each bivariate category (i.e., high for both movement and feeding site selection, or high for one and low for the other), extracting the value of several key habitat covariates at each of those points, and calculating the average covariate value across all 1000 random points within each bivariate category.

### Conflict analysis

We examined whether puma habitat selection when moving and/or feeding on kills predicts the spatial locations of human–wildlife interactions and potential for conflict using data on puma incident locations from the Washington Department of Fish and Wildlife (WDFW) predatory wildlife incidents database (https://wdfw.wa.gov/species‐habitats/living/dangerous‐wildlife/reports). We focused on records tagged as “livestock or pet injury/loss” (hereafter, “livestock conflict”; *n* = 129) and puma sightings or direct confrontations (hereafter, “sightings”; *n* = 573). We compared the predicted probability of selection from each movement‐based iSSA and feeding site RSF model at each incident location with values extracted at a set of randomly sampled background locations using binomial generalized linear models (GLM) in which the probability that a given location was an incident (1) or background (0) point was modeled as a function of the predicted movement iSSA value plus the feeding site RSF value at that location. Full details of the conflict analysis can be found in Appendix S1, Section S1: Supplementary Methods.

## RESULTS

**FIGURE 2:**
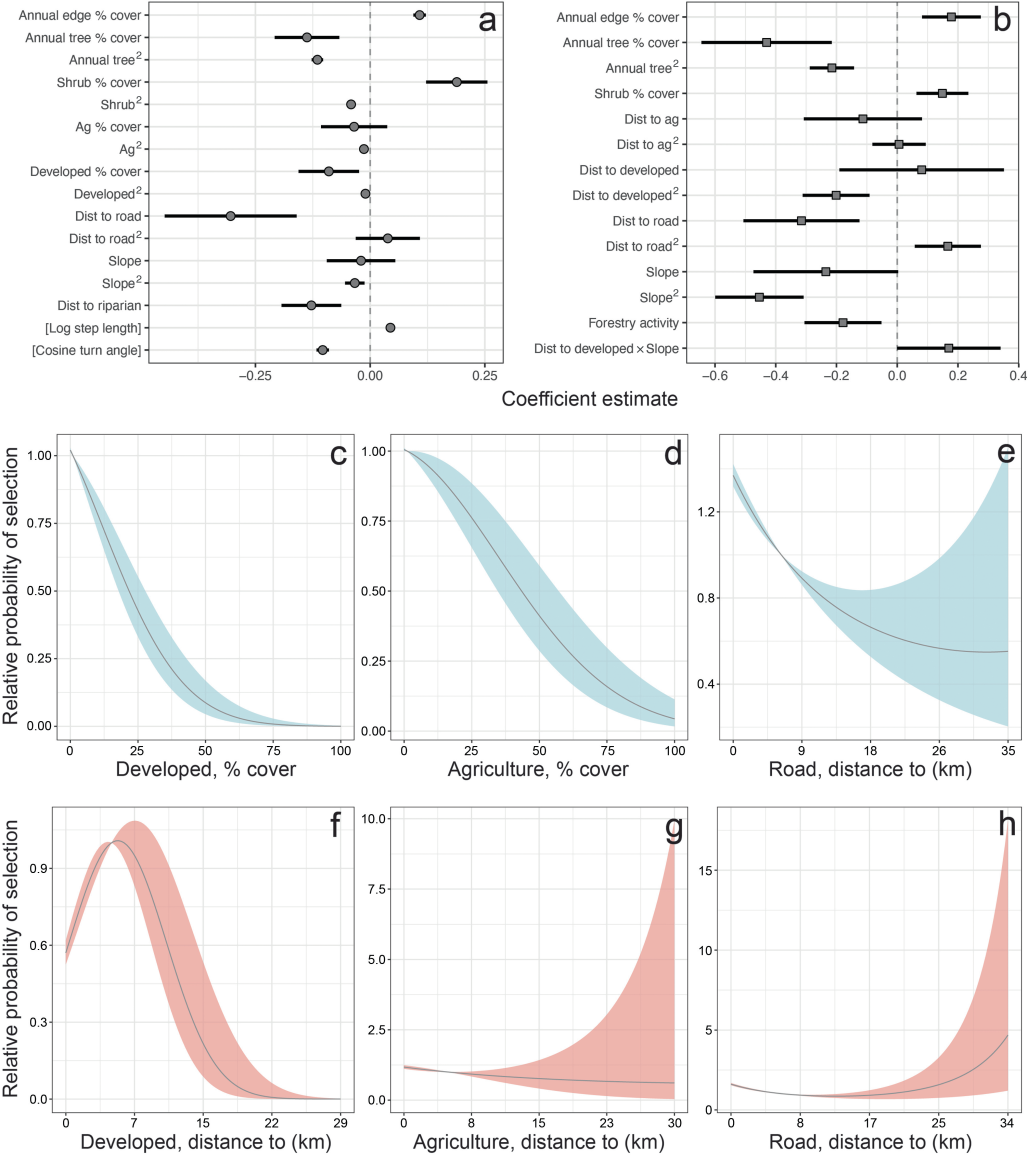
Results of movement‐based integrated step selection analyses (iSSA) and feeding site resource selection function (RSF) models using pooled datasets. Coefficient plots present parameter estimates (points) and 95% confidence intervals (bars) for the top iSSA (a) and RSF (b) models. Panels (c) through (h) highlight select habitat covariates that exhibited nonlinear relationships with relative probability of selection (*w*(*x*), see text) in the top iSSA (c–e, blue) and RSF (f–h, red) models. Lines in (c) through (h) represent the predicted value of *w*(*x*) across the observed range of the habitat covariate when all other covariates are held at their means. Shaded areas are ±1 SE. See Appendix S1: Figure S2 for additional nonlinear relationships.

Cross‐validation indicated strong predictive performance for the top pooled models (movement iSSA: *R*_*s*_ = 0.97 ± 0.03 SD; feeding site RSF: *R*_*s*_ = 0.87 ± 0.07 SD), with top age‐sex class‐specific models also exhibiting moderate to high predictive power (Appendix S1: Table S6). Model selection performed on the pooled kill dataset with domestic and “unknown” kills (*n* = 26 kill sites) removed resulted in the selection of the same top model as the full dataset and nearly identical parameter estimates (compare Figure 2b to Appendix S1: Figure S1).

Pooled models revealed that pumas selected for similar vegetation characteristics when moving and when feeding on kills, exhibiting a strong preference for forest edge habitats and moderate levels of tree cover (Figure 2a,b; Appendix S1: Figure S2a,d). However, the effect of anthropogenic land uses on habitat selection differed substantially between movement and feeding site selection models. When moving through their environment, pumas selected against areas of increasing development (Figure 2c) and agriculture cover (Figure 2d). When feeding at their kills, however, avoidance of human land uses was substantially lower, with pumas selecting for areas relatively close to development (Figure 2f) and exhibiting only weak and highly variable selection relative to agriculture (Figure 2b,g). Major roads also exerted differential influences on puma habitat use, with pumas selecting for areas closer to major roads when moving (Figure 2e) and exhibiting a weak preference for areas farther from roads when feeding on kills (Figure 2h). Finally, pumas avoided active forestry when feeding (negative “forestry activity” coefficient in Figure 2b), but this covariate was not included in our top iSSA model, suggesting that forestry activity is not a strong driver of puma habitat selection while moving. Drivers of habitat selection were relatively consistent among age‐sex classes, and class‐specific models revealed similar patterns of movement versus feeding site selection to those observed for pooled data. Vegetation characteristics (forest edge, tree cover, and/or shrub cover) were important drivers of both movement and feeding site selection for all age‐sex classes, but most classes only avoided human land use when moving (Appendix S1: Figures S3 and S4). While top movement iSSA models for all age‐sex classes included terms for development and agriculture, this was only the case for the adult female feeding site RSF, with the top feeding site RSF for all other age‐sex classes only including nonhuman covariates.

**FIGURE 3:**
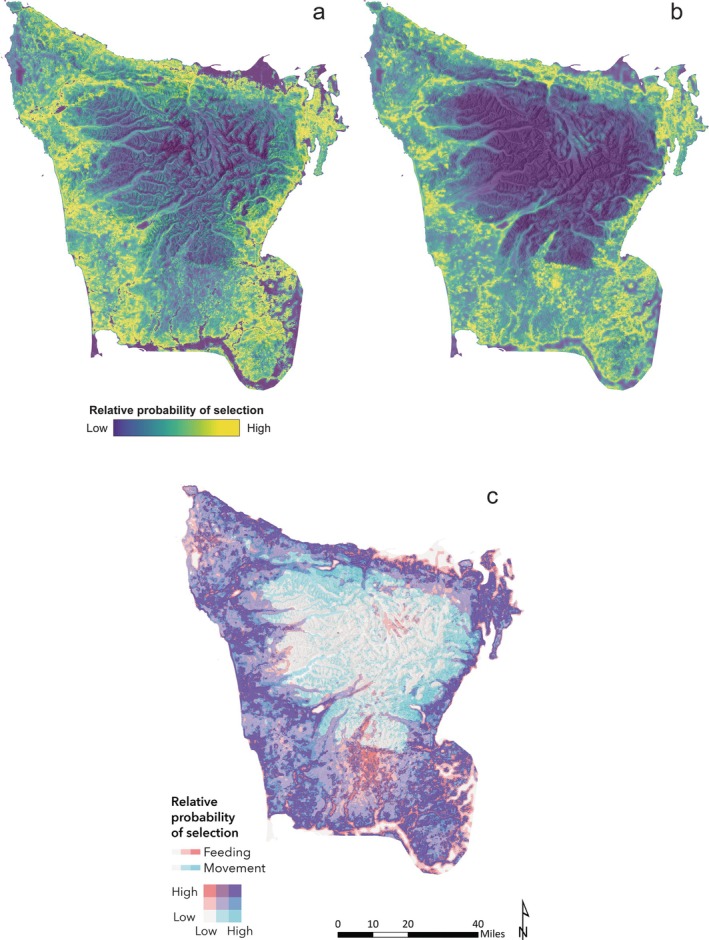
Habitat suitability (measured as relative probability of selection by pumas) for (a) movement and (b) feeding on kills across the study area in the Olympic Peninsula, Washington, USA, as predicted by the top pooled integrated step selection analyses (iSSA) and resource selection function (RSF) models, respectively. The bivariate map in (c) illustrates overlap in areas categorized as low, medium, or high suitability for movement and feeding. 1 mile = 1.6 km.

Mapping predicted habitat suitability for movement (Figure 3a) and feeding (Figure 3b) across the Olympic Peninsula revealed considerable overlap between models in areas categorized as high suitability (within the top third for predicted *w*(***x***) values across the entire study area, see Appendix S1) for both movement and feeding; 69.0% of high suitability areas for movement were categorized as high for feeding, and 74.7% of high suitability feeding areas were categorized as high for movement (Figure 3c). Overall, these “high–high” areas (purple areas in Figure 3c) constitute 24% of the study area (3900 km^2^) and reflect important habitat for satisfying multiple life‐history requirements, including hunting, dispersal, and territorial patrol/maintenance. Similarly important “high–high” areas are also mapped for each age‐sex class in Appendix S1: Figures S5–S7.

**FIGURE 4:**
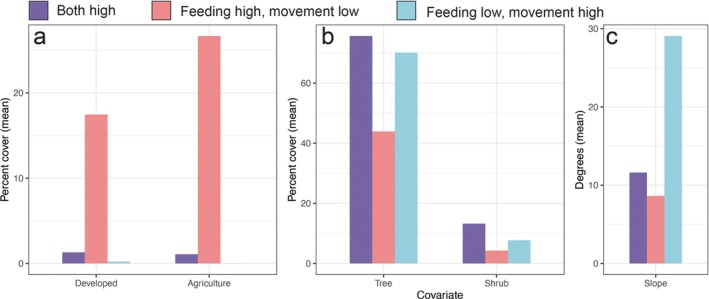
Average value of key (a) anthropogenic, (b) vegetation, and (c) terrain variables across 1000 random locations sampled from areas where both movement and feeding suitability are high (purple bars), where feeding suitability is high but movement suitability is low (red bars), and where movement suitability is high but feeding suitability is low (blue bars). The spatial distributions of these three categories of habitat suitability are shown in Figure 3c.

For pooled models, the total area of locations categorized as high suitability for movement and low suitability for feeding, or vice versa, was considerably smaller—1.7% (260 km^2^) and 2.6% (420 km^2^) of the study area, respectively (Figure 3c)—but nonetheless reflects important differences in the habitat types that our models predict as being suitable for each behavior. Sampling random locations across the study area revealed that, where only feeding suitability was high, the average percent cover of development (17.5% ± 20.0 SD %) and agriculture (26.7% ± 31.7%) was substantially greater than where movement and feeding suitability were both high (development: 1.3% ± 3.0%; agriculture: 1.1% ± 4.5%; Figure 4a), while average tree and shrub cover was lower (tree: 43.9% ± 28.3% where only feeding suitability is high vs. 75.7% ± 17.7% where both are high; shrub: 4.3% ± 10.7% vs. 13.2% ± 22.3%; Figure 4b). Where only movement suitability was high, the average slope of the terrain was substantially greater (29.1° ± 6.4°) than where both movement and feeding suitability were high (11.6° ± 5.0°; Figure 4c).

**FIGURE 5:**
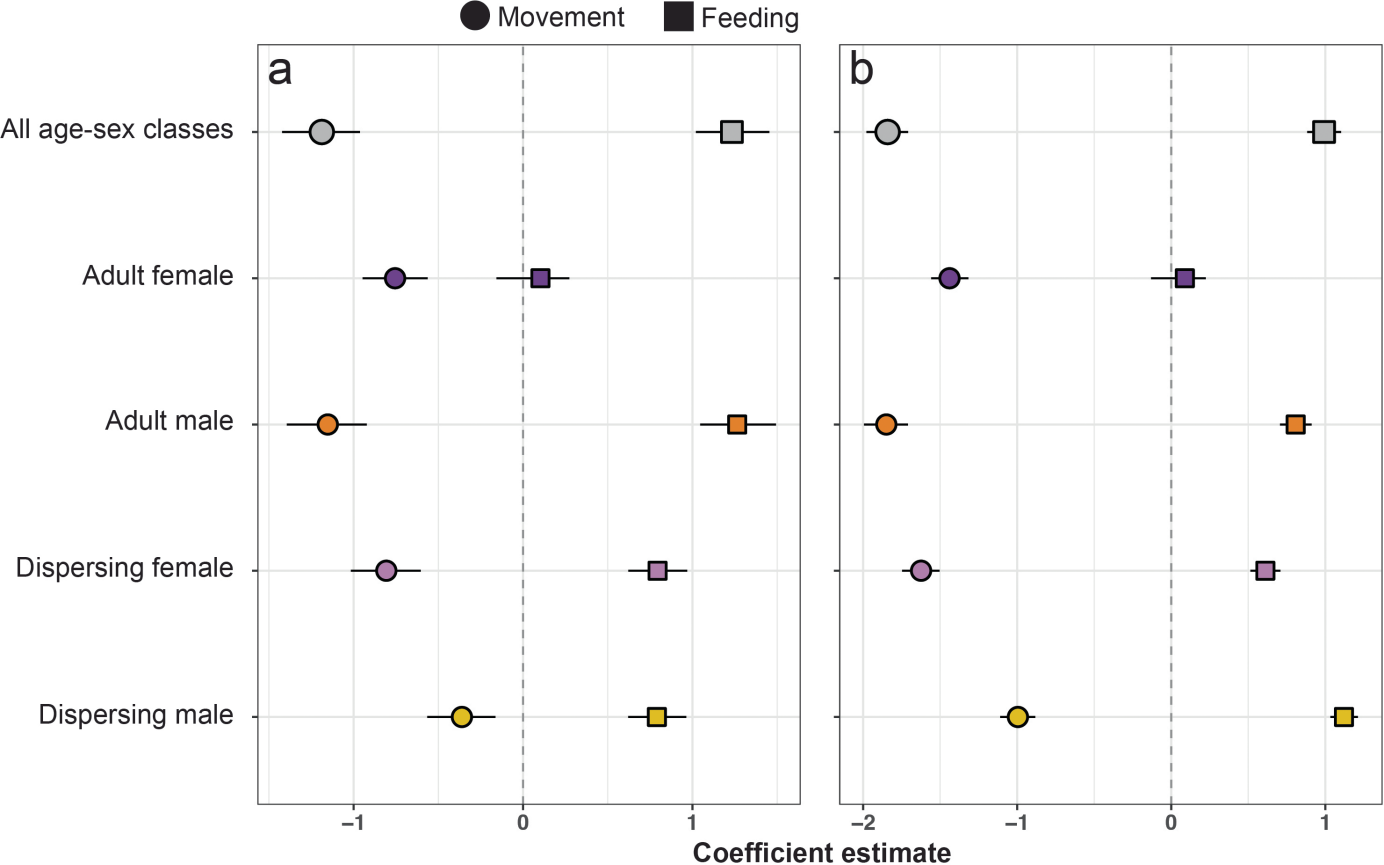
Association between puma habitat selection when moving (circles) or feeding on kills (squares) and the probability of occurrence of (a) conflict with livestock or (b) puma sightings. Points are coefficient estimates (±95% confidence intervals) from binomial generalized linear models (GLMs) modeling the probability that a given location is a conflict location as a function of movement or feeding suitability at that location. Separate models were fit to the conflict dataset using habitat suitability values from the top pooled integrated step selection analyses (iSSA) and resource selection function (RSF) models and top models for each individual age‐sex class.

When considering all age‐sex classes combined (i.e., the pooled models), habitat suitability for feeding on kills was strongly positively associated with the probability of both livestock conflict and puma sightings, while habitat suitability for movement was strongly negatively associated with both incident types (Figure 5). When considering age‐sex classes independently, feeding site suitability was positively associated with both incident types among all pumas except adult females (for whom the association between feeding site suitability and probability of livestock conflict or sightings was not significantly different from zero). In contrast, habitat suitability for movement was strongly negatively associated with the probability of livestock conflict and sightings for all age‐sex classes (Figure 5).

## DISCUSSION

Sustaining viable populations of large carnivores in increasingly human‐dominated landscapes requires conservation strategies that maintain carnivore habitat as well as coexistence strategies that minimize human–wildlife conflict. By comparing puma habitat use across age‐sex classes when engaged in two critical behaviors, this study highlights several key considerations in planning for human–carnivore coexistence. We found support for our first hypothesis (as outlined in the *Introduction*), observing that, in general, puma habitat selection while moving was more sensitive to human development and agriculture than was habitat selection when feeding at kills (Figure 2). We found modest support for our second hypothesis that dispersing individuals would be more likely than adults to use areas near people. When moving, all pumas avoided modified landscapes, but only adult female pumas avoided development and agriculture when feeding (Appendix S1: Figures S3 and S4). Additionally, male pumas, and particularly dispersing males, exhibited a preference for areas near major roads when moving, while female pumas showed weak or no selection relative to roads (Appendix S1: Figure S3). Finally, our analysis of puma–human interactions and potential for conflict revealed that puma habitat selection when feeding was strongly associated with the probability of both livestock conflict and sightings generally, and that for every age‐sex class except females, habitat selection when moving was negatively associated with both incident types for all pumas (Figure 5). These findings (which support our third but not our fourth hypotheses) indicate that pumas tend to encounter livestock, pets, and people in areas that are highly suitable for hunting native prey, but that puma movement pathways across the landscape tend to avoid areas with a high probability of encountering people or their animals. Our findings support management strategies that discourage wildlife feeding (which may attract pumas to areas near people) and promote defensive infrastructure to protect livestock from pumas in good hunting habitat.

Our analysis highlights key areas of multifunctional habitat across the Olympic Peninsula that are predicted to be of high suitability for both moving and feeding (see “high–high” regions for all pumas in Figure 3b and those identified for each age‐sex class in Appendix S1: Figure S7). By allowing pumas to satisfy multiple life‐history requirements, these areas may be particularly important for supporting the persistence of the puma population in the region. High suitability in these areas is driven in large part by vegetation cover, which prior work has shown is a key factor facilitating the use of human‐dominated landscapes by pumas and other carnivores, allowing carnivores to hunt, move, and rest while avoiding direct interactions with people (Kertson et al., 2011; Nickel et al., 2020; Suraci, Frank, et al., 2019; Sweanor et al., 2008). When moving and feeding at kills, pumas in our study preferred shrub cover, moderate tree cover, and forest edges, habitat types that may provide relatively easy movement pathways, high prey availability, good hunting cover, and concealment from people (Suraci et al., 2020). Forest edges are especially valuable habitats for pumas given elevated hunting success in these areas (Cristescu et al., 2019; Laundré & Hernández, 2003), and edge habitat was found to be important across behaviors and age‐sex classes in our study (Figure 2; Appendix S1: Figures S3 and S4). However, edges are dynamic landscape features that are often associated with human disturbance (e.g., agricultural field edges or the boundaries of the wildland–urban interface) and may draw pumas toward working or developed landscapes, potentially increasing the opportunity for conflict.

We found that pumas exhibited opposite responses to two distinct but related forms of human disturbance: developed areas and major roads. Pooled analyses showed that while pumas in general avoided developed areas while moving, they tended to select for roads and adjacent areas (Figure 2a). While the dataset used here only considered larger roadways (state routes) and did not capture the extensive logging roads associated with timber extraction (see Appendix S1, Section S1: Supplementary Methods), selection for habitat in the vicinity of major roads may reflect a preference for less energetically demanding movement routes, particularly for dispersers and male pumas that must engage in extensive movements to find new home ranges or defend large territories (Nickel et al., 2021). Indeed, male pumas in our study, especially dispersing males, selected more strongly for roads while moving than did females (Appendix S1: Figure S3). Roads inevitably lead to developed areas, and this fact may help to explain the disproportionate number of male over female pumas killed by the state agency following livestock conflict (112 males vs. 73 females statewide from 2020 to 2022; see harvest reports at https://wdfw.wa.gov/hunting/management/game‐harvest/). The disproportionate removal of males is concerning for the Olympic cougar population, where the importance of the genetic contributions of males has been highlighted in recent research (Zeller et al., 2023).

The strong association between livestock conflict and habitat selection when hunting native prey suggests that, in our study area, puma conflict with livestock may be largely opportunistic, driven by the presence of domestic animals in deer and elk habitat and puma selection for places where native ungulate hunting success is high, such as forest edges (Knopff et al., 2014; Laundré & Hernández, 2003). Work in other systems also indicates that puma predation on secondary prey (including smaller native species and domestic animals) is largely opportunistic (Alldredge et al., 2019; Cristescu et al., 2019; Nisi, Benson, & Wilmers, 2022) and that domestic animal depredations are predominantly on smaller livestock species (sheep and goats) and pets (primarily domestic cats) (Dellinger et al., 2021). Taken together, these findings suggest a dual strategy to encourage coexistence in human communities living with pumas: (1) discourage deer and elk from spending disproportionate time near people by minimizing public feeding of wildlife, and (2) keep pets indoors at night, and keep sheep and goats in roofed nighttime enclosures away from field edges and close to houses. Such strategies may be highly effective at mitigating conflict with domestic animals by reducing the opportunity for pumas to encounter these species while hunting native prey. Coexistence can be further supported by implementing nonlethal solutions following puma–livestock conflict (e.g., translocation) to keep more pumas alive and to improve the lower genetic diversity and higher inbreeding coefficients characteristic of the Olympic puma population and other puma populations adjacent to human population centers (Benson et al., 2019; Wultsch et al., 2023).

Both reported depredation incidents and puma sightings were strongly correlated with feeding site selection among all pumas except adult females (Figure 5). This may be explained by the fact that (1) many areas near human‐modified landscapes provide high‐quality deer and elk habitat and (2) pumas may spend protracted periods of time (several days; Smith et al., 2015) in the vicinity of kills, potentially increasing the opportunity for human encounters. Areas across the Olympic Peninsula that our models categorized as highly suitable for feeding (but not for movement) had on average moderate levels of human development (mean percent cover of development = 17.5%; Figure 4a). Such moderately developed areas (e.g., exurban and rural communities) have been shown in other systems to be particularly dangerous for pumas. In Colorado's Front Range (Alldredge et al., 2019) and in the Santa Cruz Mountains of California (Nisi, Benson, & Wilmers, 2022), puma‐livestock conflict was found to be highest at low‐to‐moderate housing densities, in the latter case leading to the highest levels of retaliatory killing of pumas in these areas. Benson et al. (2023) found that, across the state of California, annual survival of pumas was lowest in areas with “intermediate human presence,” a finding also driven by a higher incidence of retaliatory killings in these areas relative to both wildlands and more urbanized landscapes. By contrast, we found that livestock predation events and sightings by people were strongly negatively associated with puma habitat selection when moving, and indeed all pumas exhibited strong avoidance of agriculture and development when moving. These findings suggest that pumas are highly sensitive to the risks posed by people when moving through the landscape, likely avoiding human encounters both spatially (Nickel et al., 2021; Suraci, Clinchy, et al., 2019) and temporally (e.g., by increasing nocturnality; Gaynor et al., 2018, Nickel et al., 2020), but that pumas are willing to accept higher anthropogenic risk when hunting, which may be related to hunger levels and time since their last kill (Blecha et al., 2018; Hooten et al., 2019).

It is worth noting that the degree of selection for a given habitat factor can vary depending on the spatiotemporal scale at which selection is assessed (Nisi, Suraci, et al., 2022; Suraci, Frank, et al., 2019). Given the datasets available here (i.e., two‐hour movement relocations and discrete locations of kills) and the methods appropriate for their analysis, the movement and feeding site selection models necessarily addressed selection at different scales and were developed using different approaches to assess available habitat. The iSSA model used to address movement‐based selection compared used and available habitat at the scale of a two‐hour movement “step,” with average step length being 594 (±732 SD) m, while the feeding site RSF compared used kill locations to random locations drawn from across a given puma's area of use (i.e., MCP “home range”), which could conceivably take multiple days to traverse. Furthermore, the conditional model used to fit the iSSA compared each used step to a unique set of 10 available steps within a movement kernel, and thus the available habitat differed with each step, while in the RSF model, the set of available locations was the same for each used location. For these reasons, predictions from the iSSA and RSF models are not interchangeable and only comparable in a relative sense (i.e., locations predicted to be of relatively high suitability for movement vs. feeding). However, we considered it important to match the scale of analysis to the scale at which pumas are likely making relevant behavioral decisions, and assumed that the decision of where to move to next while traversing the landscape occurs on an inherently smaller scale than the decision of where in the home range to hunt for prey. Our results should therefore be interpreted with the understanding that the appropriate spatial scale and availability domain are inherently different between our movement and feeding analyses.

### Conclusion

Stable and resilient coexistence between wildlife and humans requires co‐adaptation, that is, behavioral adjustments by both humans and wildlife to minimize conflict and potentially achieve mutual benefits (Carter & Linnell, 2023; Lute & Carter, 2020). Pumas have shown a remarkable capacity to adapt to human environments, taking advantage of modified landscapes and anthropogenic resources, and in many cases persisting in close proximity to large human population centers (Benson et al., 2019; Kertson et al., 2011; Moss et al., 2016). Human adaptation to puma presence, however, has arguably been less positive. Puma hunting limits have recently been increased across much of the western USA, negatively impacting puma populations (Erwin et al., 2024), and lethal removal of “problem” individuals remains the primary response to conflict (Alldredge et al., 2019; Dellinger et al., 2021). These actions set up a scenario that Carter and Linnell (2023) refer to as “reciprocal damages,” in which each party (humans and pumas) has real or perceived negative impacts on the other, and which is inconsistent with long‐term coexistence. However, our work contributes to the growing body of evidence suggesting a feasible pathway toward stable human–puma coexistence wherein (i) sufficient high‐quality puma habitat is retained in and near human‐dominated landscapes, and (ii) achievable adjustments to human behavior are promoted to reduce opportunistic encounters between pumas and domestic animals. Advancing such outcomes will require additional efforts to quantify and communicate the advantages that predators can provide to human societies (e.g., through their ability to limit ungulate prey where potential conflict with ungulates is high Gilbert et al., 2017, 2021), ideally moving toward a “sustained co‐benefits” scenario, a coexistence state characterized by strong co‐adaptation and recognition of mutual benefit (Carter & Linnell, 2023).

## CONFLICT OF INTEREST STATEMENT

The authors declare no conflicts of interest.

## Supporting information


Appendix S1.

## Data Availability

Nonsensitive data and model outputs (Suraci, 2025) are available in Figshare at https://doi.org/10.6084/m9.figshare.29594576.v1. Code (Lacey & Suraci, 2025) is available in Zenodo at https://doi.org/10.5281/zenodo.16240598. GPS location data on puma movement and prey data on the Olympic Peninsula, Washington, USA, are sensitive and not available publicly; these data are owned by Panthera, the Lower Elwha Klallam Tribe, the Quinault Indian Nation, the Skokomish Tribe, the Makah Tribe, and the Point No Point Treaty Council, and available to qualified researchers by contacting the Director of the Puma Program at Panthera (info@panthera.org) and requesting data collected as part of the “Olympic Cougar Project” from 2017 to 2022 (note that Panthera will solicit the approval of all collaborators and respond accordingly).
