# Supplementary material for: Puma habitat preferences when moving and feeding predict the potential for human–carnivore conflict in shared landscapes
Source: Ecol Appl. 2025 Sep 11;35(6):e70101. doi: 10.1002/eap.70101 (PMC12426584; doi:10.1002/eap.70101)
Supplement: Supplementary file 1 — Appendix S1. [file EAP-35-e70101-s001.pdf]

# Appendix S1: Supplementary Materials

## Puma habitat preferences when moving and feeding predict the potential for human–carnivore conflict in shared landscapes

Justin P. Suraci, L. Mae Lacey, Patrick T. Freeman, Andrew Stratton, Caitlin Kupar, Kimberly Sager-Fradkin, Dylan Bergman, Bethany Ackerman, Kristen A. Phillips, Shannon Murphie, Cassandra Sullivan, L. Mark Elbroch

### SECTION S1: SUPPLEMENTARY METHODS

**Puma GPS collaring, data cleaning, and identification of dispersal events**

We captured pumas using trailing hounds, and immobilized them with 1.5 cc/45 kg of BAM (per ml, 27.3 mg Butorphanol Tartrate, 9.1 mg Azaperone Tartrate, and 10.9 mg Medetomidine HCL) following safety guidelines reviewed and described in the University of Idaho IACUC Protocols IACUC-2020-15 and IACUC-2017-79. Pumas were monitored every 5 minutes for temperature, respiration and heart rate, and they were reversed with an equal dosage of atipamezole as they were given BAM + 0.5 ml naltrexone.

We performed data cleaning on an initial GPS collar dataset from 93 individual pumas collared between January 1, 2017 and September 29, 2022. We first truncated all movement tracks by removing the first and last 12 hours to exclude erroneous locations taken when the collar was on but not deployed on the animal. Using the *bayesmove* package in R (Cullen et al. 2022) we then calculated step lengths, turning angles, and net squared displacement (NSD) between each GPS location for each puma, where NSD is equal to the squared distance between the first location of a puma track and each subsequent location. We plotted NSD vs. time to identify clusters of points substantially removed from all other points for a given animal and visually inspected movement tracks to determine whether these points were also associated with periods when collars were active but not on an animal. If so, these locations were removed. We imposed a consistent two-hour GPS sampling rate across all pumas and collar deployments by excluding periods during which the fix rate was greater than two hours and thinning datasets with shorter fix rates (i.e., one-hour).

Given the focus of the present analysis on age and sex class-specific habitat selection, we endeavored to segment each puma movement track into those points associated with each of three age classes: dependent young, disperser, and adult. Several pumas (n = 44) were known to be adults at first capture and their entire movement track was therefore classified as “adult”. Other animals in our dataset (n = 31) were collared as dependent young and continuously monitored throughout dispersal and, in some cases, into adulthood. For these individuals, we identified dispersal start and end dates from field records where available (e.g., when field biologists recorded the approximate date that an animal left it’s mother, or date of death during incomplete dispersal) and otherwise used the NSD-based modeling approach

described by Bastille-Rousseau et al. (2016), which attempts to identify transitions between latent, coarse-scale movement states (e.g., dispersal, nomadism, home ranging) using a first order Markovian process. We fit latent state models to NSD data from pumas originally collared as dependent young using the *lsmnsd* package in R (Bastille-Rousseau et al. 2016) and identified the start and/or end dates of dispersal events as model-determined transition points between coarse-scale movement states associated with home ranging vs. long-distance movements. All locations before the dispersal start date were classed as “dependent young” and all locations after the dispersal end date were classed as “adult”. We retained only those individuals and locations that could be confidently categorized as “disperser” or “adult”, resulting in a final dataset consisting of 63 unique individuals, nine of which were collared during both disperser and adult life stages. Final sample sizes for all age and sex classes were: adult female, n = 26 individuals; adult male, n = 19; dispersing female, n = 9; dispersing male, n = 18.

**Model covariate development**

Our non-human covariates included both vegetation and terrain features. We acquired annual estimates of percent tree cover as yearly, 30-m resolution rasters from the Rangeland Analysis Platform (RAP; Allred et al. 2021). We derived annual forest edge habitat from tree cover by first defining forest as all pixels with a percent tree cover of 45% or more (McIntosh et al. 2009, Shennan-Farpón et al. 2021) and then calculating the distance from each forested cell to the closest non-forested cell. All forest pixels with a distance of 60-m (i.e., two pixel widths) or less from a non-forest pixel were then classified as edge habitat. The three remaining non-human covariates were only available for a single time point and thus were not prepared annually. Shrub cover (30-m resolution) was derived from the 2019 National Land Cover Database (NLCD; Dewitz and U.S. Geological Survey 2021) “shrub/scrub” category. To quantify the effects of terrain and its resulting impacts on movement capacity, hunting cover, etc, we used the ALOS World 3D digital surface model (30-m resolution; Tadono et al. 2014) to calculate three terrain measures known to affect puma habitat selection and movement: (i) slope; (ii) ruggedness (via the vector ruggedness measure or VRM; Sappington et al. 2007); and (iii) the topographic position index (TPI), which captures the degree to which the area surrounding a given location consists of topographic features such as ridgelines, valley bottoms, or sloping terrain (Dickson and Beier 2007, Nickel et al. 2021, Nisi et al. 2022). We quantified the euclidean distance from each location on the landscape to the nearest riparian area (U.S. Environmental Protection Agency 2021).

We developed six human covariates that captured anthropogenic activity and landscape impacts. Developed areas were identified by combining the “low-”, “medium-”, and “high-intensity development” land cover categories from the 2019 NLCD. We identified agricultural lands as the union of two datasets: all pixels classified as “pasture/hay” or “cultivated crops” in the 2019 NLCD, and pixels classified as “cropland” circa 2016 in a high-resolution (10-m) dataset on agricultural land cover developed as a component of the American Farmland Trust’s Farms Under Threat analysis (https://csp-fut.appspot.com/) (CSP 2020). These layers were used to calculate percent cover of development and agriculture within various radii (as described in detail below) and we also calculated the euclidean distance from each location on the landscape to the nearest development or agriculture pixel. Washington DoT’s state routes dataset (Washington State Department of Transportation 2023) was

used to quantify the euclidean distance from each location on the landscape to the nearest major state roadways.

Finally, we developed a "forestry activity" layer, representing pixels within our study area experiencing active forestry during the study period (2018 - 2022), by drawing on three data layers: the Washington State Forest Practice Applications (FPA) dataset (Washington State Department of Natural Resources 2017), the U.S. Forest Service Timber Harvest (TH) dataset (U.S. Forest Service 2016), and Global Forest Change (GFC) dataset developed by Hansen et al. (2013a). The FPA and TH polygon datasets provide the boundaries and date ranges for approved forestry leases/harvest units on state and federal lands, respectively, and we subsetted these datasets to just those units with date ranges overlapping our study period. However not all approved harvest units are actively being logged. We therefore overlaid these approved harvest unit polygons with all areas identified by the GFC as having experienced forest loss between 2018 and 2021. Any FPA and TH polygons with no detectable forest loss according to the GFC were removed from our active forestry layer, with two exceptions: (1) harvest units with completion dates during 2022 were retained given that GFC data was not available for this year at the time of analysis; and (2) all TH units with a completion date during our study were retained, given that USFS thinning practices may not necessarily be captured by the GFC, but still represent substantial disturbance on the ground. Remaining active forestry polygons were rasterized at 30 m such that all pixels within an active polygon were coded as 1. All covariate preparation was conducted in Google Earth Engine (GEE; Gorelick et al. 2017), and final covariate layers were projected to 'NAD83 UTM Zone 10N' and exported at 30-m resolution.

**Model selection, validation, and inference**

We employed a two-stage model fitting approach for both movement and feeding site models. For each dataset (pooled, age-sex class-specific) and model type (iSSA, RSF), we first fit two separate sets of models consisting of various combinations of either human land use covariates ('human models') or natural land cover and terrain covariates ('non-human models'; see Tables S2 to S5) based on a priori hypotheses, and used AICc to identify the top human and non-human model (Stage 1). In all cases, Pearson coefficients for correlations between all pairs of covariates included in the same model were < 0.6. In a second modeling stage, we then combined the covariates from the top human and non-human models for a given dataset into up to three 'combined' models consisting of (1) all covariates occurring in the top human and non-human models, (2) all influential covariates occurring in either Stage 1 top model (i.e., those with standard error estimates not crossing zero), if this differed from combined model 1, and (3) all influential covariates plus a term for the interaction between human development (either percent cover or distance to, depending on the top human model) and slope. The latter was added given evidence from other study systems that pumas will move through steeper terrain when in proximity to developed areas (Nisi et al. 2022b). Finally, for each dataset, we compared the top human, top non-human, and all combined models using AICc and considered the model with the lowest AICc value to be the final model for that dataset.

For the final model in each dataset, we used k-fold cross validation to estimate model predictive performance. For both model types (RSF and iSSA) we trained models that included all covariates in a

given final model on a randomly selected 80% subset of the data and calculated predictions from that model for the remaining 20% test set. For RSFs, our approach followed that described by Boyce et al. (2002) and consisted of (1) binning predicted values into 10 quantiles, (2) quantifying the number of times that the predicted value for used points fell into each quantile, and (3) calculating the Spearman rank correlation coefficient ($R_s$) between the rank of each quantile bin (1 to 10) and the number of used locations in that bin. For iSSA models we followed the approach described by Fortin et al. (2009, see also Dancose et al. 2011). For each stratum in the test set, used and available steps were ranked from 1 to 11 based on predicted value, and we calculated $R_s$ between rank number and the number of times the used step in a stratum received each rank. The above process was repeated multiple times for each final model (50 replicates per model for RSF, 30 replicates for iSSA given substantially longer model run times), and we calculated the mean and SD of $R_s$ values (range = [0,1]) as our estimate of model performance. Values closer to one represent better predictive performance. As a comparison, we also calculated $R_s$ values expected under random habitat selection (i.e., if models have no predictive power), following Fortin et al. (2009). Random selection is expected to produce $R_s$ values close to zero.

**Conflict analysis**

We acquired all dangerous incident locations recorded between December 2016 and August 2022 and filtered these to retain records of "livestock or pet injury/loss" (hereafter, "livestock conflict"; n = 129) and puma sightings or direct confrontations (hereafter, "sightings"; n = 573). As noted above, while puma sightings are not necessarily a source of conflict (and could even be considered by some to be positive opportunities to interact with nature), we included these incidents given their potential role in public perceptions of the risk of sharing the landscape with a large carnivore. As our primary focus was on the potential for perceived conflict/risk, we retained all incident reports regardless of whether they had been investigated and confirmed by WDFW. This included 107 confirmed and 22 unconfirmed livestock conflict incidents and 182 confirmed and 391 unconfirmed sightings incidents.

We determined the relative probability that a puma would select a conflict location when moving or feeding at kills by extracting the $w(\boldsymbol{x})$ values from iSSA and RSF prediction surfaces at each conflict location. We then compared model predictions for relative probability of selection at conflict locations to those at random 'background' locations across the study area. In an effort to only sample background points from areas with some potential for conflict, we focused background point sampling on areas near human settlements or working landscapes and outside of large natural areas (such as Olympic National Park). To do so we first identified all agricultural pixels across the study area (using the same definition of agricultural landscapes as employed above, i.e., "pasture/hay" or "cultivated crops" pixels from the 2019 NLCD, and "cropland" pixels from the Farms Under Threat analysis) as well as any pixels with high levels of nighttime light, indicative of human habitation. For the latter, we took the average radiance value for each pixel in the study area across all monthly nighttime light images collected in 2021 by the Visible Infrared Imaging Radiometer Suite (VIIRS) instrument aboard NASA-NOAA's Suomi NPP satellite and considered any pixel with an average radiance value ≥ 0.6 to represent a developed area. This cut-off was chosen to capture areas of relatively rural/dispersed development (identified from satellite imagery) and was high enough to exclude radiance from bare rock and snow in high altitude areas. Using nighttime light to identify development rather than pixels classified as developed by the 2019 NLCD allowed us to

exclude roads through otherwise undeveloped areas, which are occasionally coded as developed in NLCD. Agriculture and nighttime light pixels were then buffered by 3 km (a buffer radius large enough to capture all livestock conflict points in our dataset) to derive our final sampling domain for background points. For each conflict dataset (livestock and sightings) we sampled 10 background points for every conflict point. We then extracted the average value of each habitat selection model prediction surface (i.e., movement iSSA and feeding site RSF from pooled models and age-sex class-specific models) with a 100-m radius around each background and conflict point.

For each conflict dataset, we fit a series of five binomial generalized linear models (GLM) - one each for each puma age-sex class and the pooled data set - in which the probability that a given location was a conflict (1) or background (0) point was modeled as a function of the predicted movement iSSA value plus the feeding site RSF value at that location, with iSSA and RSF values extracted from the appropriate puma dataset. Before fitting models, predictor data were scaled by subtracting the mean and dividing by the standard deviation. All models were fit in R, and we identified the statistical significance of a predictor variable by evaluating whether its 95% confidence interval overlapped zero.

## SECTION S2: SUPPLEMENTARY TABLES AND FIGURES

**Table S1.** Habitat covariates included in the movement-based iSSA and/or feeding site RSF habitat selection models. 'Sub-model' indicates whether a given covariate was used in the 'non-human' or 'human' sub-models during two-stage model selection. The scale of effect columns provide, for relevant data layers, the scale that single-variable models indicated was most influential in driving habitat selection. See main text for details on sub-model development and scales of effect.

| **Covariate** | **Sub-model** | **Source** | **Scale of effect, movement iSSA (150 m or 1 km)** | **Scale of effect, feeding RSF (100m, 500m, 1km)** |
|---|---|---|---|---|
| Tree, percent cover | Non-human | Rangeland Analysis Platform, v3[1] | 1 km | 1 km |
| Forest edge, percent cover | Non-human | Rangeland Analysis Platform, v3 | 150 m | 100 m |
| Shrub, percent cover | Non-human | National Land Cover Database (NLCD) 2019[2] | 150 m | 500 m |
| Elevation | Non-human | ALOS World 3D (AW3D) global digital surface model[3] | 150 m | 100 m |
| Riparian, distance to | Non-human | EPA Riparian Zones 2019[4] | -- | -- |
| Slope | Non-human | AW3D | (Along path) | 1 km |
| Ruggedness | Non-human | AW3D | (Along path) | -- |
| Terrain position index (TPI) | Non-human | AW3D | (Along path) | -- |
| Agriculture, percent cover | Human | NLCD 2019 & CSP (2020) | 150 m | 100 m |
| Agriculture, distance to | Human | NLCD 2019 & CSP (2020) | -- | -- |
| Development, percent cover | Human | NLCD 2019 | 150 m | 1 km |
| Development, distance to | Human | NLCD 2019 | -- | -- |
| Road, distance to | Human | Washington DOT[5] | -- | -- |
| Forestry activity, percent cover | Human | Washington State Forest Practice Applications[6], USFS Timber Harvests[7], & Hansen et al. (2013b) | 1 km | -- |

[1](Allred et al. 2021); [2](Dewitz and U.S. Geological Survey 2021); [3](Tadono et al. 2014); [4](U.S. Environmental Protection Agency 2021); [5](Washington State Department of Transportation 2023); [6](Washington State Department of Natural Resources 2017); [7](U.S. Forest Service 2016)

**Table S2.** Model selection results for **stage one** of the two-stage model selection process for ***movement-based iSSA models*** used to select the top human (H1-H10) and non-human (NH1-NH8) sub models. Values shown for each age-sex class and the pooled data set (all age-sex classes combined) are ΔAICc scores. NA indicates that a model failed to converge. The top model for each age-sex class and sub-model type has a ΔAICc score of 0.

| **Candidate model** | | **Pooled** | **Adult female** | **Adult male** | **Disperser female** | **Disperser male** |
|---|---|---|---|---|---|---|
| *HUMAN SUB-MODELS* | | | | | | |
| H1 | ag cover + dev cover + road dist | 170.90 | 33.20 | 70.18 | 22.22 | 56.48 |
| H2 | ag dist + dev dist + road dist | 393.08 | 70.10 | 181.30 | 58.83 | 96.63 |
| H3 | ag cover + dev cover + road dist + forestry | 149.61 | 14.50 | 58.55 | 23.61 | 54.45 |
| H4 | ag dist + dev dist + road dist + forestry | NA | 81.49 | 172.33 | 59.56 | 87.46 |
| H5 | ag cover + ag cover^2 + dev cover + dev cover^2 + road dist + road dist^2 | ***0.00*** | ***0.00*** | ***0.00*** | 0.13 | 0.05 |
| H6 | ag dist + ag dist^2 + dev dist + dev dist^2 + road dist + road dist^2 + forestry | 392.42 | 39.27 | 166.61 | 62.26 | 92.15 |
| H7 | ag cover + ag cover^2 + dev cover + dev cover^2 | 49.12 | 19.81 | 42.67 | ***0.00*** | 4.30 |
| H8 | ag dist + ag dist^2 + dev dist + dev dist^2 | 390.28 | 56.31 | 177.50 | 60.77 | 104.63 |
| H9 | ag cover + ag cover^2 + dev cover + dev cover^2 + road dist + dev cover * night | 0.43 | 0.56 | 7.46 | 2.58 | ***0.00*** |
| H10 | ag cover + ag cover^2 + dev cover + dev cover^2 + dev cover * night | 49.41 | 17.40 | 43.25 | 1.59 | 6.14 |
| *NON-HUMAN SUB-MODELS* | | | | | | |
| NH1 | slope + TPI + tree cover + tree cover^2 + shrub cover + shrub cover^2 + riparian dist | 139.30 | 77.47 | 25.02 | 32.71 | 106.09 |
| NH2 | slope + slope^2 + edge cover + tree cover + tree cover^2 + shrub cover + shrub cover^2 + riparian dist | ***0.00*** | ***0.00*** | ***0.00*** | ***0.00*** | ***0.00*** |
| NH3 | slope + slope^2 + edge cover + tree cover + tree cover^2 + shrub cover + shrub cover^2 | 266.84 | 6.33 | 223.33 | 20.85 | 24.11 |
| NH4 | slope + edge cover + tree cover + shrub cover | 796.58 | 173.37 | 607.32 | 22.44 | 94.16 |
| NH5 | TPI + edge cover + tree cover + shrub cover | 784.11 | 163.80 | 597.51 | 43.44 | 87.90 |
| NH6 | slope + TPI + edge cover + tree cover + shrub cover + riparian dist | 421.99 | 264.09 | NA | 3.13 | NA |
| NH7 | slope + slope^2 + edge cover + tree cover + shrub cover | NA | 162.56 | 575.94 | 24.28 | 60.90 |
| NH8 | edge cover + tree cover + tree cover^2 + shrub cover + shrub cover^2 | 301.51 | 37.64 | 278.03 | 43.77 | 45.67 |

**Table S3.** Model selection results for **stage two** of the two-stage model selection process for ***movement-based iSSA models*** use to select the final model from among the top human and non-human models (corresponding to those shown in Table S2) as well as two or more 'combo' models combining covariates from the human and non-human models. **Table continues on next page*

| **Candidate model** | | **Delta AIC** |
|---|---|---|
| *POOLED (ALL AGE-SEX CLASSES)* | | |
| Top human (H5) | ag cover + ag cover^2 + dev cover + dev cover^2 + road dist + road dist^2 | 2635.57 |
| Top non-human (NH2) | slope + slope^2 + edge cover + tree cover + tree cover^2 + shrub cover + shrub cover^2 + riparian dist | 425.75 |
| Pooled combo 1 | ag cover + ag cover^2 + dev cover + dev cover^2 + road dist + road dist^2 + slope + slope^2 + edge cover + tree cover + tree cover^2 + shrub cover + shrub cover^2 + riparian dist | ***0.00*** |
| Pooled combo 2 | ag cover + ag cover^2 + dev cover + dev cover^2 + road dist + edge cover + tree cover + tree cover^2 + shrub cover + shrub cover^2 | 379.63 |
| *ADULT FEMALE* | | |
| Top human (H5) | ag cover + ag cover^2 + dev cover + dev cover^2 + road dist + road dist^2 | 796.28 |
| Top non-human (NH2) | slope + slope^2 + edge cover + tree cover + tree cover^2 + shrub cover + shrub cover^2 + riparian dist | 75.09 |
| AF combo 1 | ag cover + ag cover^2 + dev cover + dev cover^2 + road dist + road dist^2 + slope + slope^2 + edge cover + tree cover + tree cover^2 + shrub cover + shrub cover^2 + riparian dist | 1.67 |
| AF combo 2 | ag cover + ag cover^2 + dev cover + dev cover^2 + road dist + road dist^2 + slope + slope^2 + edge cover + tree cover + tree cover^2 + shrub cover + shrub cover^2 | 0.98 |
| AF combo 3 | ag cover + ag cover^2 + dev cover + dev cover^2 + road dist + road dist^2 + slope + slope^2 + edge cover + tree cover + tree cover^2 + shrub cover + shrub cover^2 + dev cover * slope | ***0.00*** |
| *ADULT MALE* | | |
| Top human (H5) | ag cover + ag cover^2 + dev cover + dev cover^2 + road dist + road dist^2 | 1461.18 |
| Top non-human (NH2) | slope + slope^2 + edge cover + tree cover + tree cover^2 + shrub cover + shrub cover^2 + riparian dist | 205.32 |
| AM combo 1 | ag cover + ag cover^2 + dev cover + dev cover^2 + road dist + road dist^2 + slope + slope^2 + edge cover + tree cover + tree cover^2 + shrub cover + shrub cover^2 + riparian dist | 12.93 |
| AM combo 2 | ag cover + ag cover^2 + dev cover + dev cover^2 + road dist + road dist^2 + slope + slope^2 + edge cover + tree cover + tree cover^2 + shrub cover + shrub cover^2 + riparian dist + dev cover * slope | ***0.00*** |
| *DISPERSER FEMALE* | | |
| Top human (H7) | ag cover + ag cover^2 + dev cover + dev cover^2 | 177.86 |

| **Candidate model** | | **Delta AIC** |
|---|---|---|
| Top non-human (NH2) | slope + slope^2 + edge cover + tree cover + tree cover^2 + shrub cover + shrub cover^2 + riparian dist | 41.08 |
| DF combo 1 | ag cover + ag cover^2 + dev cover + dev cover^2 + slope + slope^2 + edge cover + tree cover + tree cover^2 + shrub cover + shrub cover^2 + riparian dist | 0.96 |
| DF combo 2 | ag cover + ag cover^2 + dev cover + dev cover^2 + slope + edge cover + tree cover + tree cover^2 + shrub cover + shrub cover^2 + riparian dist | ***0.00*** |
| DF combo 3 | ag cover + ag cover^2 + dev cover + dev cover^2 + slope + edge cover + tree cover + tree cover^2 + shrub cover + shrub cover^2 + riparian dist + dev cover * slope | 1.42 |
| *DISPERSER MALE* | | |
| Top human (H9) | ag cover + ag cover^2 + dev cover + dev cover^2 + road dist + dev cover * night | 300.21 |
| Top non-human (NH2) | slope + slope^2 + edge cover + tree cover + tree cover^2 + shrub cover + shrub cover^2 + riparian dist | 50.15 |
| DM combo 1 | ag cover + ag cover^2 + dev cover + dev cover^2 + road dist + road dist^2 + slope + slope^2 + edge cover + tree cover + tree cover^2 + shrub cover + shrub cover^2 + riparian dist | 5.96 |
| DM combo 2 | ag cover + ag cover^2 + dev cover + dev cover^2 + road dist + slope + slope^2 + edge cover + tree cover + tree cover^2 + riparian dist | ***0.00*** |

**Table S4.** Model selection results for **stage one** of the two-stage model selection process for ***feeding site RSF models*** used to select the top human (H1-H10) and non-human (NH1-NH8) sub models. Values shown for each age-sex class and the pooled data set (all age-sex classes combined) are ΔAICc scores. The top model for each age-sex class and sub-model type has a ΔAICc score of 0. **Table continues on next page*

| **Candidate model** | | **Pooled** | **Adult female** | **Adult male** | **Disperser female** | **Disperser male** |
|---|---|---|---|---|---|---|
| *HUMAN SUB-MODELS* | | | | | | |
| H1 | ag cover + dev cover + road dist | 115.27 | 140.39 | 32.46 | 23.92 | 16.14 |
| H2 | ag dist + dev dist + road dist | 21.37 | 75.37 | 2.55 | 12.21 | 8.75 |
| H3 | ag cover + dev cover + road dist + forestry | 117.23 | 145.88 | 42.89 | 35.37 | 14.97 |
| H4 | ag dist + dev dist + road dist + forestry | 21.61 | 84.08 | 12.83 | 23.22 | 2.99 |
| H5 | ag cover + ag cover^2 + dev cover + dev cover^2 + road dist + road dist^2 | 98.93 | 133.28 | 33.20 | 16.60 | 6.72 |
| H6 | ag dist + ag dist^2 + dev dist + dev dist^2 + road dist + road dist^2 + forestry | ***0.00*** | 13.33 | 10.80 | 22.43 | ***0.00*** |
| H7 | ag cover + ag cover^2 + dev cover + dev cover^2 | 154.07 | 129.60 | 73.30 | 9.73 | 27.53 |
| H8 | ag dist + ag dist^2 + dev dist + dev dist^2 | 10.02 | ***0.00*** | ***0.00*** | ***0.00*** | 14.11 |
| H9 | ag cover + ag cover^2 + dev cover + dev cover^2 + road dist | 99.63 | 133.48 | 33.36 | 29.48 | 7.59 |
| H10 | ag dist + ag dist^2 + dev dist + dev dist^2 + forestry | 7.13 | 7.09 | 8.08 | 8.32 | 9.52 |
| H11 | ag cover + ag cover^2 + dev cover + dev cover^2 + forestry | 160.78 | 135.73 | 81.40 | 17.64 | 29.46 |
| H12 | ag cover + ag cover^2 + dev cover + dev cover^2 + road dist + road dist^2 + forestry | 99.08 | 138.63 | 43.92 | 31.22 | 3.77 |
| *NON-HUMAN SUB-MODELS* | | | | | | |
| NH1 | slope + tree cover + tree cover^2 + shrub cover + shrub cover^2 + riparian dist | 60.33 | 20.56 | 1.34 | 9.83 | 48.16 |
| NH2 | slope + slope^2 + edge cover + tree cover + tree cover^2 + shrub cover + shrub cover^2 + riparian dist | 0.30 | 11.02 | 1.50 | 19.04 | 11.11 |
| NH3 | slope + slope^2 + edge cover + tree cover + tree cover^2 + shrub cover + shrub cover^2 | 8.65 | ***0.00*** | 6.38 | 14.11 | ***0.00*** |
| NH4 | slope + edge cover + tree cover + shrub cover | 107.68 | 52.59 | 20.67 | 16.60 | 36.02 |
| NH5 | edge cover + tree cover + shrub cover | 177.09 | 121.02 | 91.67 | 20.76 | 32.45 |
| NH6 | slope + edge cover + tree cover + shrub cover + riparian dist | 100.14 | 54.24 | 8.86 | 9.29 | 36.96 |
| NH7 | slope + slope^2 + edge cover + tree cover + shrub cover | 55.55 | 26.56 | 16.57 | 17.46 | 0.07 |

| Candidate model | | Pooled | Adult female | Adult male | Disperser female | Disperser male |
|---|---|---|---|---|---|---|
| NH8 | edge cover + tree cover + tree cover^2 + shrub cover + shrub cover^2 | 130.42 | 49.46 | 50.38 | 1.57 | 29.21 |
| NH9 | tree cover + tree cover^2 + shrub cover + shrub cover^2 + riparian dist | 126.93 | 47.92 | 32.01 | ***0.00*** | 43.24 |
| NH10 | edge cover + tree cover + shrub cover + shrub cover^2 + riparian dist | 156.31 | 90.10 | 43.49 | 9.51 | 39.51 |
| NH11 | slope + slope^2 + edge cover + tree cover + tree cover^2 + shrub cover + riparian dist | ***0.00*** | 10.60 | ***0.00*** | 17.24 | 9.46 |
| NH12 | slope + tree cover + shrub cover + riparian dist | 134.66 | 54.17 | 18.50 | 17.61 | 60.67 |

**Table S5.** Model selection results for **stage two** of the two-stage model selection process for ***feeding site RSF models*** use to select the final model from among the top human and non-human models (corresponding to those shown in Table S4) as well as two or more 'combo' models combining covariates from the human and non-human models. **Table continues on next page*

| **Candidate model** | | **Delta AIC** |
|---|---|---|
| *POOLED (ALL AGE-SEX CLASSES)* | | |
| Top human (H6) | ag dist + ag dist^2 + dev dist + dev dist^2 + road dist + road dist^2 + forestry | 259.87 |
| Top non-human (NH11) | slope + slope^2 + edge cover + tree cover + tree cover^2 + shrub cover + riparian dist | 57.00 |
| Pooled combo 1 | ag dist + ag dist^2 + dev dist + dev dist^2 + road dist + road dist^2 + forestry + slope + slope^2 + edge cover + tree cover + tree cover^2 + shrub cover + riparian dist | 3.51 |
| Pooled combo 2 | ag dist + ag dist^2 + dev dist + dev dist^2 + road dist + road dist^2 + forestry + slope + slope^2 + edge cover + tree cover + tree cover^2 + shrub cover | 1.95 |
| Pooled combo 3 | ag dist + ag dist^2 + dev dist + dev dist^2 + road dist + road dist^2 + forestry + slope + slope^2 + edge cover + tree cover + tree cover^2 + shrub cover + dev dist * slope | ***0.00*** |
| *ADULT FEMALE* | | |
| Top human (H8) | ag dist + ag dist^2 + dev dist + dev dist^2 | 74.20 |
| Top non-human (NH3) | slope + slope^2 + edge cover + tree cover + tree cover^2 + shrub cover + shrub cover^2 | 79.11 |
| AF combo 1 | ag dist + ag dist^2 + dev dist + dev dist^2 + slope + slope^2 + edge cover + tree cover + tree cover^2 + shrub cover + shrub cover^2 | 2.10 |
| AF combo 2 | ag dist + ag dist^2 + dev dist + dev dist^2 + slope + slope^2 + edge cover + tree cover + tree cover^2 + shrub cover + shrub cover^2 + dev dist * slope | ***0.00*** |
| *ADULT MALE* | | |
| Top human (H8) | ag dist + ag dist^2 + dev dist + dev dist^2 | 44.47 |
| Top non-human (NH11) | slope + slope^2 + edge cover + tree cover + tree cover^2 + shrub cover + riparian dist | ***0.00*** |
| AM combo 1 | ag dist + ag dist^2 + dev dist + dev dist^2 + slope + slope^2 + edge cover + tree cover + tree cover^2 + shrub cover + riparian dist | 6.17 |
| AM combo 2 | ag dist + ag dist^2 + dev dist + dev dist^2 + slope + slope^2 + edge cover + tree cover + tree cover^2 + shrub cover | 7.96 |
| AM combo 3 | ag dist + ag dist^2 + dev dist + dev dist^2 + slope + slope^2 + edge cover + tree cover + tree cover^2 + shrub cover + dev dist * slope | 8.82 |
| *DISPERSER FEMALE* | | |
| Top human (H8) | ag dist + ag dist^2 + dev dist + dev dist^2 | 16.96 |
| Top non-human (NH9) | tree cover + tree cover^2 + shrub cover + shrub cover^2 + riparian dist | ***0.00*** |

| **Candidate model** | | **Delta AIC** |
|---|---|---|
| DF combo 1 | ag dist + ag dist^2 + dev dist + dev dist^2 + tree cover + tree cover^2 + shrub cover + shrub cover^2 + riparian dist | 10.56 |
| DF combo 2 | ag dist + ag dist^2 + dev dist + dev dist^2 + tree cover + tree cover^2 + shrub cover + shrub cover^2 | 4.45 |
| DF combo 3 | ag dist + ag dist^2 + dev dist + dev dist^2 + tree cover + tree cover^2 + shrub cover + shrub cover^2 + slope + dev dist * slope | 30.50 |
| *DISPERSER MALE* | | |
| Top human (H6) | ag dist + ag dist^2 + dev dist + dev dist^2 + road dist + road dist^2 + forestry | 53.96 |
| Top non-human (NH3) | slope + slope^2 + edge cover + tree cover + tree cover^2 + shrub cover + shrub cover^2 | ***0.00*** |
| DM combo 1 | ag dist + ag dist^2 + dev dist + dev dist^2 + road dist + road dist^2 + forestry + slope + slope^2 + edge cover + tree cover + tree cover^2 + shrub cover + shrub cover^2 | 17.97 |
| DM combo 2 | ag dist + ag dist^2 + dev dist + dev dist^2 + road dist + road dist^2 + forestry + slope + slope^2 + edge cover + tree cover + tree cover^2 + shrub cover + shrub cover^2 + dev dist * slope | 15.36 |

**Table S6.** K-fold cross validation results for the top models for each model type and age-sex class (including the pooled data set consisting of data from all age-sex classes). Values are the mean (SD) of Spearman rank correlation coefficients $R_s$ across multiple iterations. $R_s$ values closer to one indicate better predictive performance. We also calculated $R_s$ values under random habitat selection ($R_s$ Random), which are expected to be close to 0. See main text for details.

| | Movement iSSA | | Feeding site RSF | |
|---|---|---|---|---|
| **Dataset** | **$R_s$** | **$R_s$ Random** | **$R_s$** | **$R_s$ Random** |
| Pooled | 0.97 (0.03) | -0.09 (0.39) | 0.87 (0.07) | -0.07 (0.36) |
| Adult female | 0.96 (0.07) | -0.1 (0.37) | 0.64 (0.14) | 0.03 (0.32) |
| Adult male | 0.97 (0.02) | -0.21 (0.31) | 0.84 (0.09) | 0.09 (0.34) |
| Disperser female | 0.84 (0.15) | 0.08 (0.32) | 0.62 (0.19) | -0.05 (0.34) |
| Disperser male | 0.92 (0.05) | 0.04 (0.29) | 0.81 (0.11) | 0.10 (0.34) |

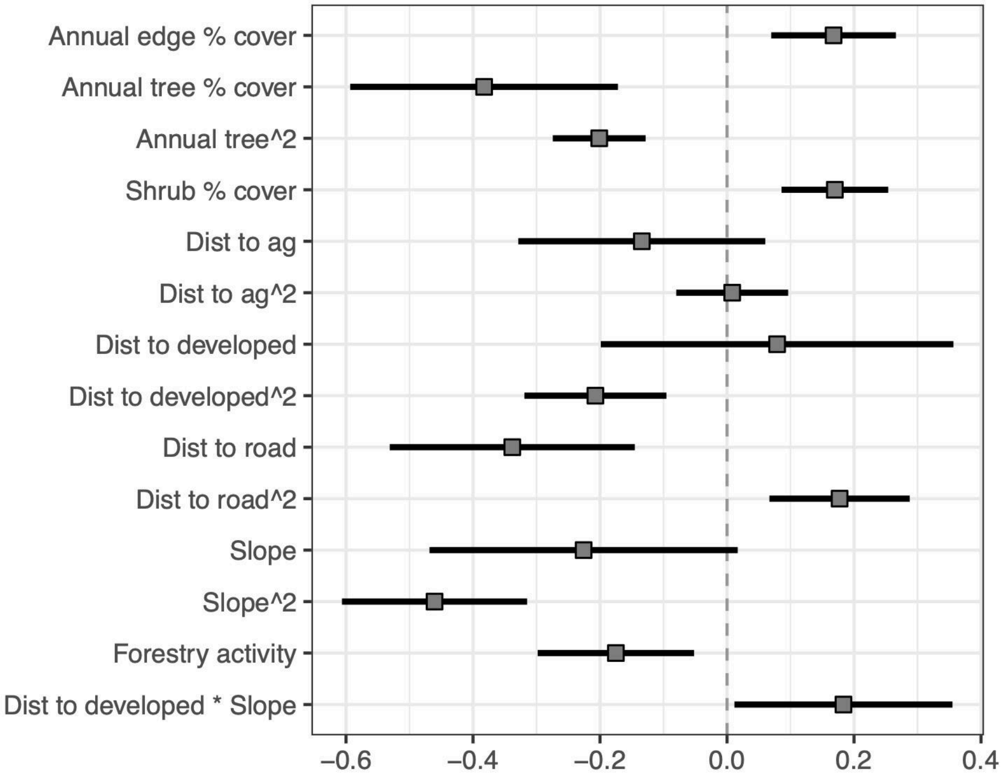


**Figure S1.** Coefficient plot from the top feeding site selection RSF model fit to data from all puma age-sex classes pooled but excluding domestic and "unknown" kills (n = 26 kill sites). Points and lines are parameter estimates and 95% confidence intervals, respectively, from the top model. Comparing this plot with Fig. 2c confirms that the inclusion of a small number of domestic and unknown kills (<2.5% of total kills) in the analysis presented in the main text did not meaningfully affect our results.

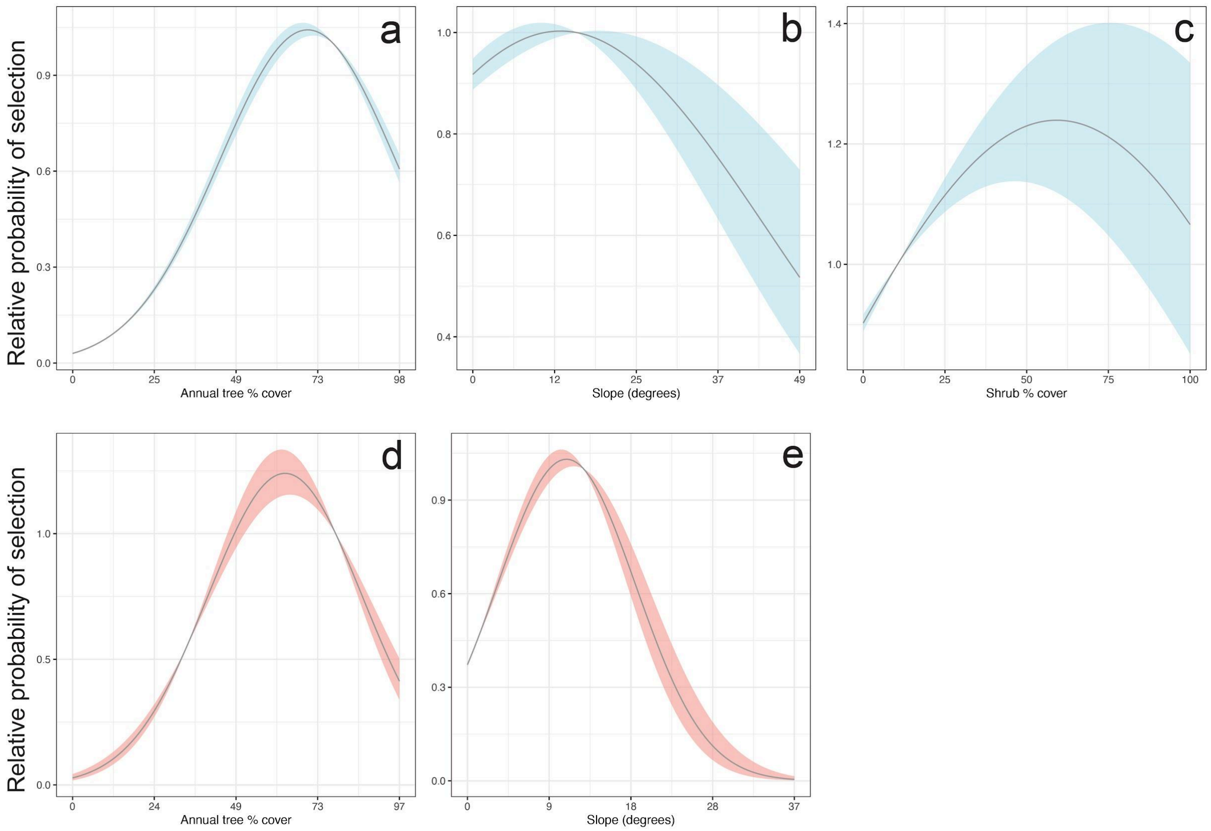


**Figure S2.** Habitat covariates included in the top pooled movement iSSA model (blue, panels **a-c**) and the top pooled feeding site RSF model (red, panels **d** and **e**) that exhibited a non-linear relationship with relative probability of selection. Lines represent the predicted relative probability of selection (*w(**x**),* see main text) across the observed range of the habitat covariate when all other covariates are held at their means. Shaded areas are ± 1 SE. The habitat covariates shown here are all those included in the top iSSA and RSF models that were not presented in Figure 2 of the main text.

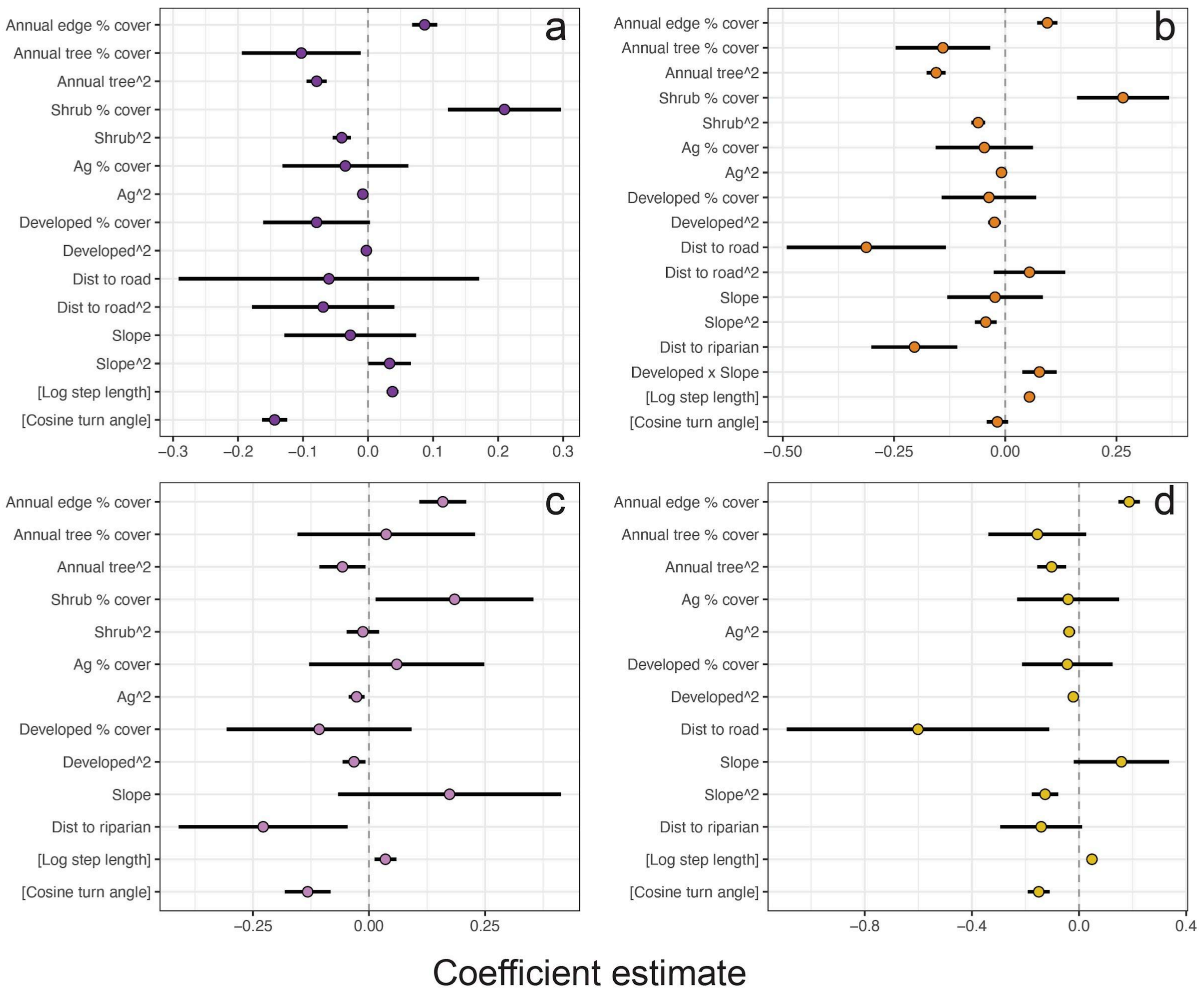


**Figure S3.** Results of the top age-sex class-specific movement iSSA models. Coefficient plots present parameter estimates (points) and 95% confidence intervals (bars) for the top model fit to movement data from (**a**) adult females, (**b**) adult males, (**c**) disperser females, and (**d**) disperser males.

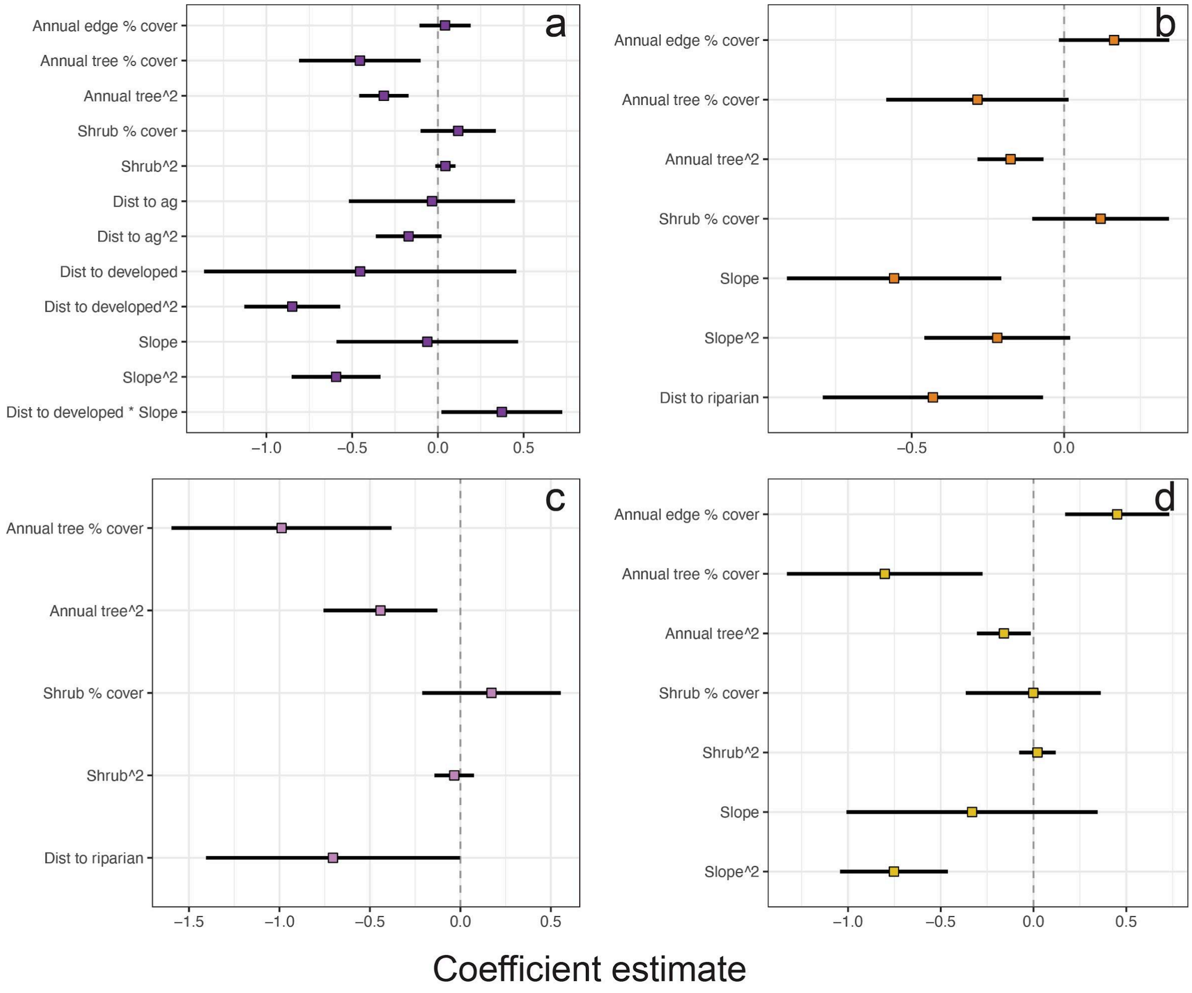


**Figure S4.** Results of the top age-sex class-specific feeding site RSF models. Coefficient plots present parameter estimates (points) and 95% confidence intervals (bars) for the top model fit to kill site data from (**a**) adult females, (**b**) adult males, (**c**) disperser females, and (**d**) disperser males.

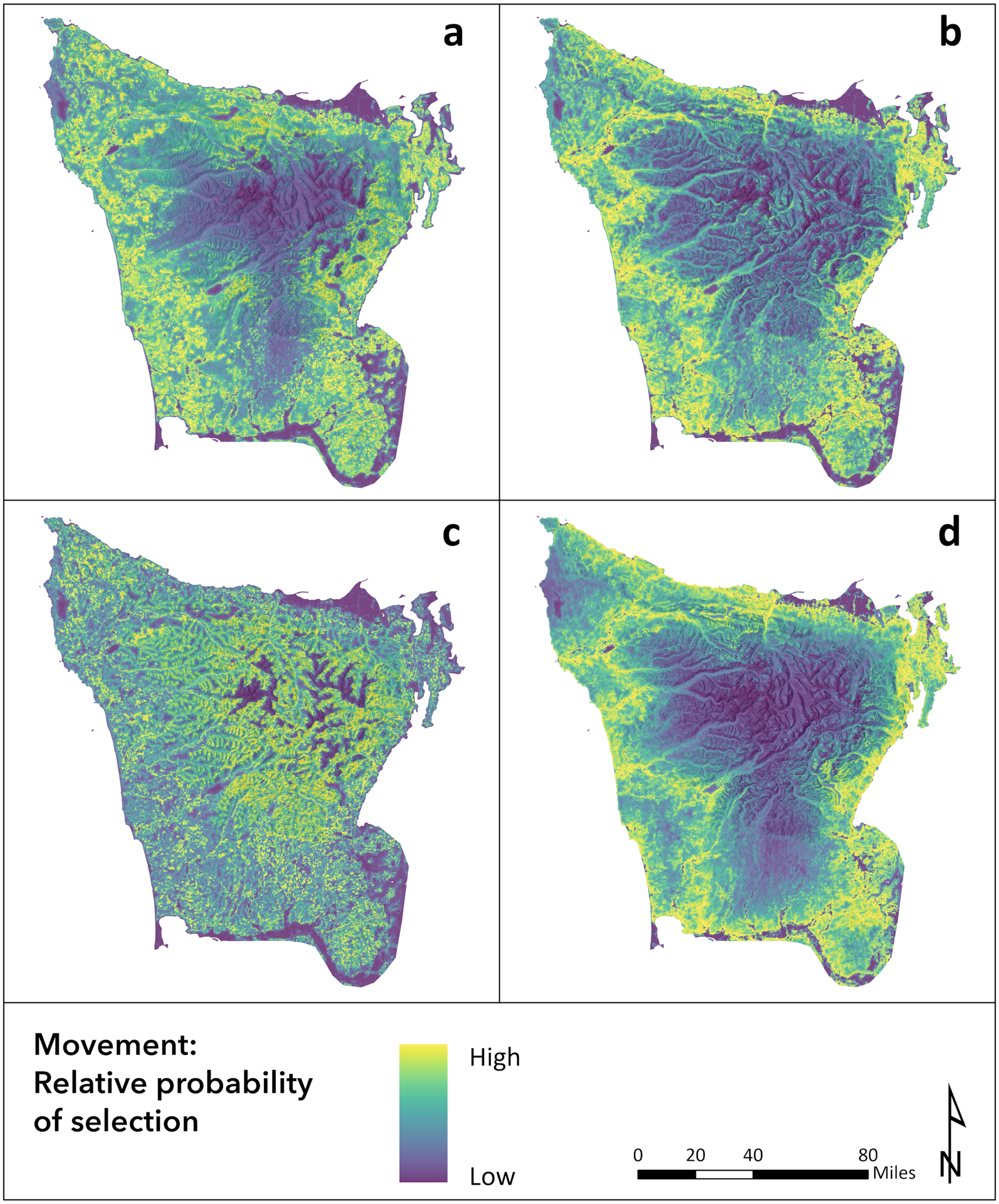


**Figure S5.** Habitat suitability (measured as relative probability of selection by pumas) for movement across the study area in the Olympic Peninsula, Washington, USA, as predicted by the top age-sex class-specific iSSA models. Maps show movement habitat suitability for (**a**) adult females, (**b**) adult males, (**c**) disperser females, and (**d**) disperser males. Note: 1 mile = 1.6 km.

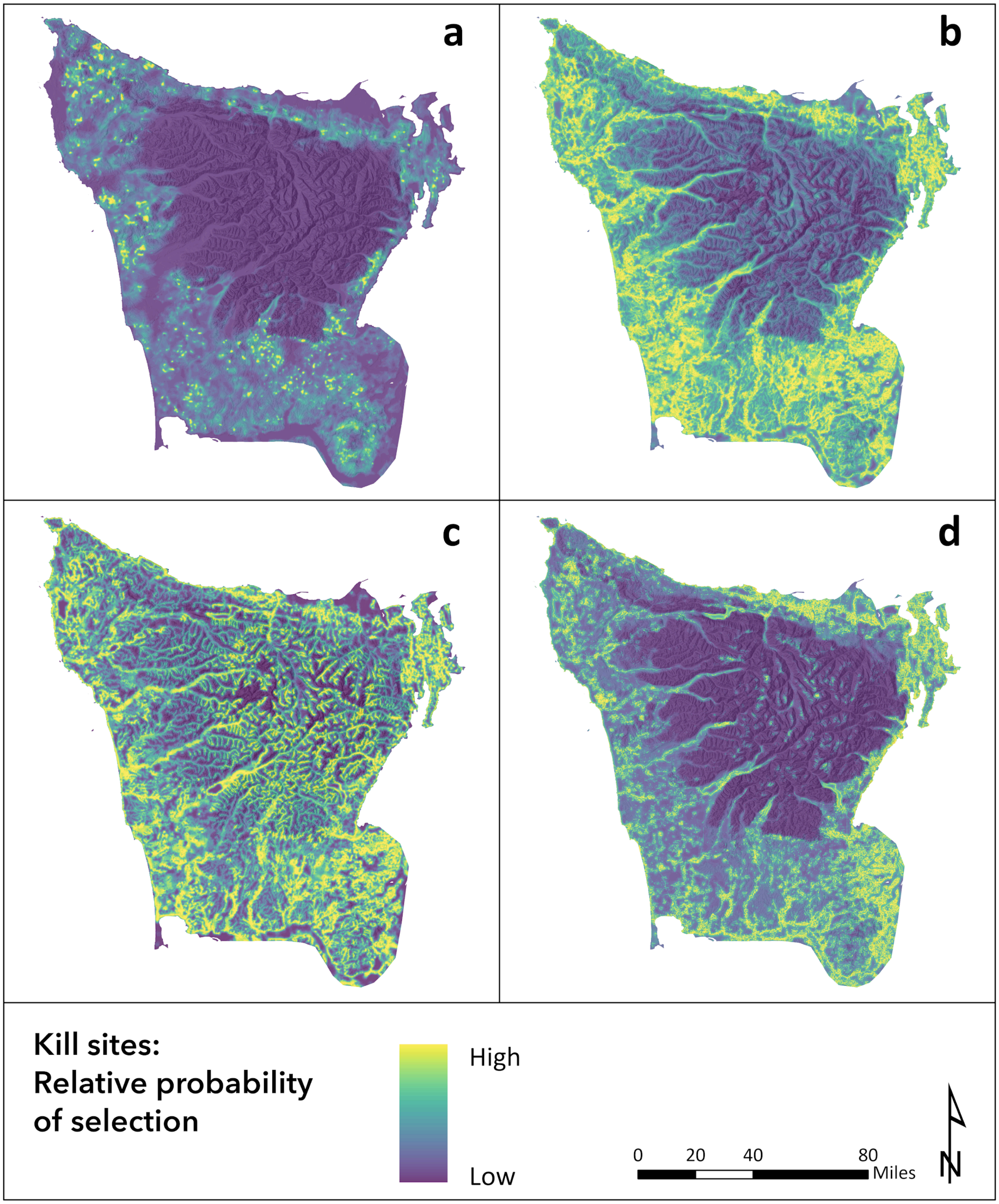


**Figure S6.** Habitat suitability (measured as relative probability of selection by pumas) for feeding at kills across the study area in the Olympic Peninsula, Washington, USA, as predicted by the top age-sex class-specific RSF models. Maps show feeding habitat suitability for (**a**) adult females, (**b**) adult males, (**c**) disperser females, and (**d**) disperser males. Note: 1 mile = 1.6 km.

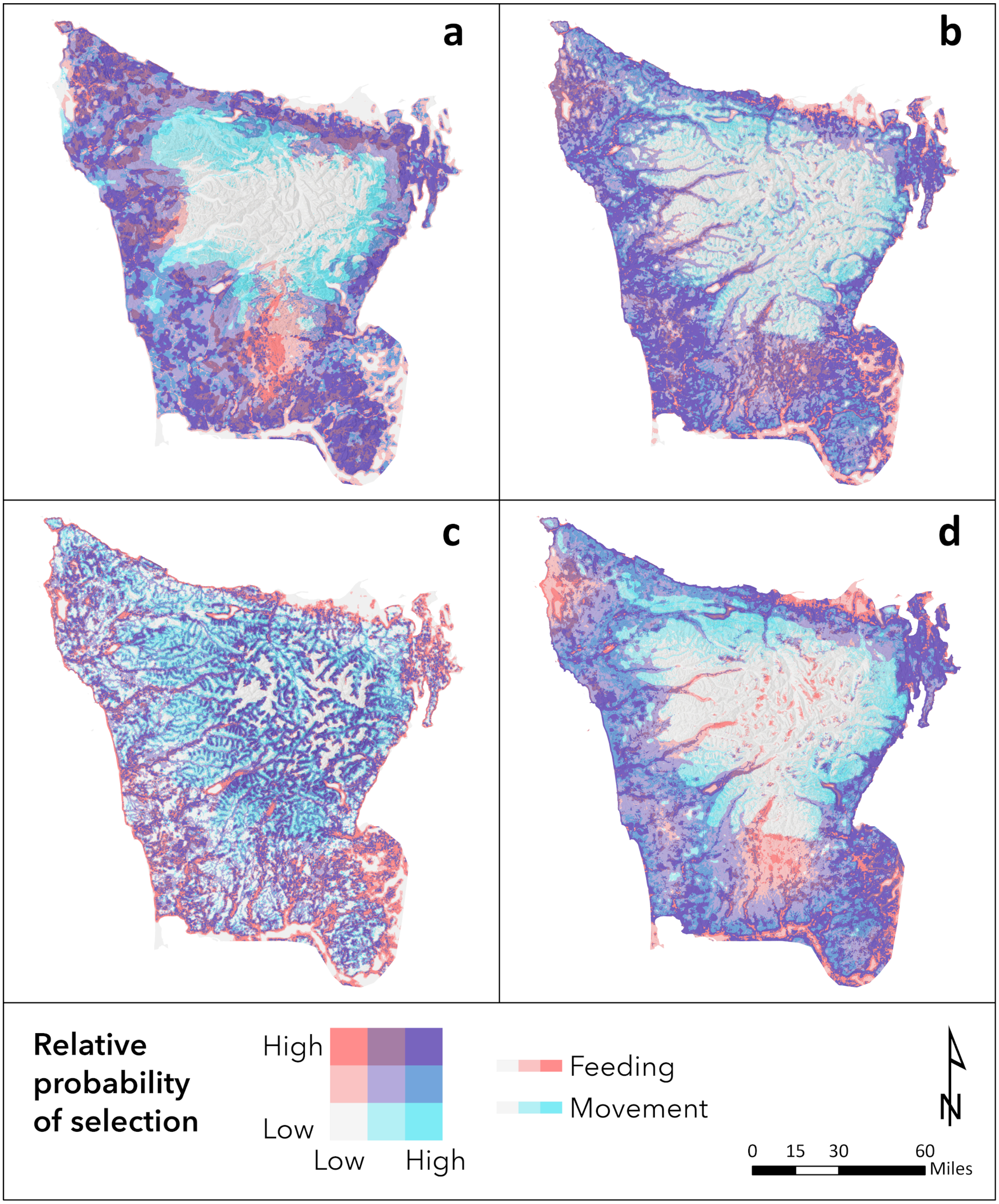


**Figure S7.** Bivariate maps showing the overlap between areas categorized as low, medium, or high suitability (i.e., in the lower, middle, or upper 33% of *w(**x**)* values across the study area, see main text) for movement (from top age-sex class-specific iSSA models) and feeding (from top age-sex class-specific RSF models). Overlap is shown for (**a**) adult females, (**b**) adult males, (**c**) disperser females, and (**d**) disperser males. Note: 1 mile = 1.6 km.

## SECTION S3: VALIDATING THE iSSA PREDICTION SURFACE

As described in the main text, developing spatially explicit predictions from step selection models is complicated by the fact that the distribution of available locations is non-static and dependent on an animal's current location (Fieberg et al. 2021). Signer et al. (2017) note that, while it is still common to predict from iSSA-type models via exponentiation of coefficient estimates from the fitted model (i.e., via Equation 1 in the main text), this can lead to overestimation of individual home range sizes and underestimation of local space use intensity. However, in the present study, we are interested in the degree to which this approach to spatial model prediction from an iSSA can serve as a useful estimate of habitat suitability for movement. We tested this by refitting the top pooled iSSA model (see Table S3 above) to data from 80% of individual pumas in our data set (n = 50 pumas, including all age and sex classes) and using the population level $\beta$ coefficients to develop a prediction surface via Equation 1 (Fig. S8). When fitting and predicting from the model for the subset of individuals, we used the same set of used and available locations (for those 50 individuals) and the same model fitting and prediction procedure as described above for the models presented in the main text.

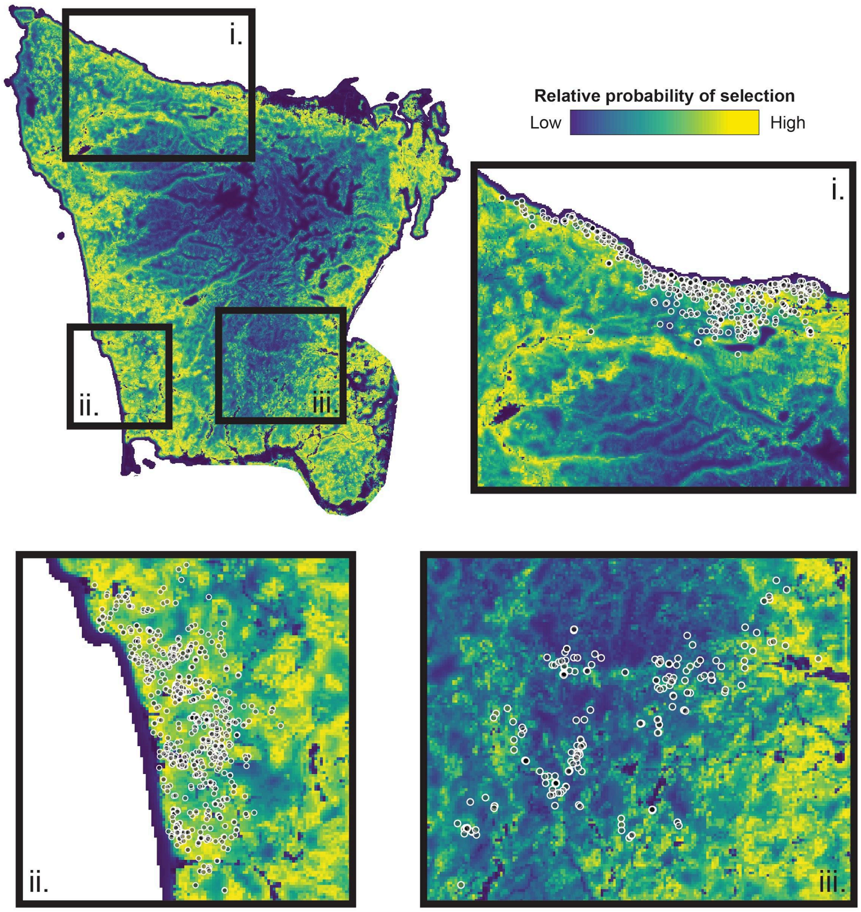


**Figure S8.** Predicted habitat suitability (relative probability of selection) from the iSSA model fit to data from 80% of individual pumas in our data set. Insets show details of the full map overlaid with used locations from out-of-sample pumas not included in the dataset used to fit the model (i: Omar, ii: Jolene, iii: Pika; see Table S7)

We then extracted the habitat suitability value from the prediction surface at each location used by the 20% of individuals (n = 13 pumas) whose data were withheld from the model and summarized the percentage of used locations falling into high (top 33.3% of habitat suitability values across the entire study area), medium (middle 33.3%) and low (bottom 33.3%) suitability areas, as predicted by Equation 1 (Table S7). These summaries provide an estimate of how well the prediction surface captures movement habitat use by out-of-sample individuals. Overall, 67.3% of out-of-sample used locations occurred within high suitability areas, while only 4.2% fell within low suitability habitat, indicating that generating spatial predictions of suitability from exponentiated iSSA model coefficients performs reasonably well in capturing puma movement-based habitat use. However, the proportion of used locations falling within high suitability habitat differed substantially between individuals (Table S7). Adult pumas tended to have the large majority of used locations in high suitability habitat (e.g., 86.5% for Jolene, 72.9% for Omar; see panels i and ii in Fig. S8), while the individuals with the lowest proportion of used locations in high suitability habitat were dispersers (Pika: 12.2% of locations in high suitability areas; Lilu during dispersal: 27.7%). This latter finding is not unexpected, as dispersing individuals moving through unfamiliar landscapes are known to use lower suitability habitat relative to the population as a whole (Elliot et al. 2014, Abrahms et al. 2017), which was indeed our motivation in developing separate habitat selection models and predictions for dispersing individuals (see, e.g., Figs. S5 and S6). However, even for these dispersers, use of the lowest suitability category was still limited (Pika: 14.3% of used locations in low suitability areas; Lilu during dispersal: 18.3%).

**Table S7.** Proportion of used locations occurring in each habitat suitability category (as predicted by the iSSA model fit to data from an 80% subset of individuals) for each puma whose data was withheld from the model.

| | | | | % Relocations in suitability category | | |
|---|---|---|---|---|---|---|
| **Name** | **Sex** | **Age class** | **Locations (n)** | **Low** | **Med** | **High** |
| Apollo | Male | Adult | 3420 | 0.6% | 25.5% | 73.9% |
| Archie | Male | Disperser | 537 | 0.7% | 34.1% | 65.2% |
| Didi* | Female | Adult | 674 | 0.0% | 22.4% | 77.6% |
| Didi* | Female | Disperser | 289 | 0.3% | 9.3% | 90.3% |
| Edgar | Male | Disperser | 344 | 4.7% | 33.1% | 62.2% |
| Georgia | Female | Adult | 77 | 3.9% | 63.6% | 32.5% |
| Hana | Female | Disperser | 150 | 0.0% | 12.0% | 88.0% |
| Jolene | Female | Adult | 786 | 1.0% | 12.5% | 86.5% |
| Kingsley | Male | Adult | 686 | 1.7% | 22.4% | 75.8% |
| Lilu* | Female | Adult | 421 | 6.4% | 31.1% | 62.5% |
| Lilu* | Female | Disperser | 1139 | 18.3% | 54.0% | 27.7% |
| Omar | Male | Adult | 734 | 2.7% | 24.4% | 72.9% |
| Pika | Female | Disperser | 238 | 14.3% | 73.5% | 12.2% |
| Rue | Female | Disperser | 326 | 17.8% | 10.7% | 71.5% |
| Vlad | Male | Disperser | 80 | 7.5% | 31.3% | 61.3% |
| ***Overall*** | | | ***9901*** | ***4.2%*** | ***28.5%*** | ***67.3%*** |

*Same individual monitored as a disperser and adult

**References**

Abrahms, B., S. C. Sawyer, N. R. Jordan, J. W. McNutt, A. M. Wilson, and J. S. Brashares. 2017. Does wildlife resource selection accurately inform corridor conservation? Journal of Applied Ecology 54:412–422.

Allred, B. W., B. T. Bestelmeyer, C. S. Boyd, C. Brown, K. W. Davies, M. C. Duniway, L. M. Ellsworth, T. A. Erickson, S. D. Fuhlendorf, T. V. Griffiths, V. Jansen, M. O. Jones, J. Karl, A. Knight, J. D. Maestas, J. J. Maynard, S. E. McCord, D. E. Naugle, H. D. Starns, D. Twidwell, and D. R. Uden. 2021. Improving Landsat predictions of rangeland fractional cover with multitask learning and uncertainty. Methods in Ecology and Evolution 12:841–849.

Bastille-Rousseau, G., J. R. Potts, C. B. Yackulic, J. L. Frair, E. H. Ellington, and S. Blake. 2016. Flexible characterization of animal movement pattern using net squared displacement and a latent state model. Movement Ecology 4:1–12.

Boyce, M. S., P. R. Vernier, S. E. Nielsen, and F. K. A. Schmiegelow. 2002. Evaluating resource selection functions. Ecological Modelling 157:281–300.

CSP. 2020. Description of the approach, data, and analytical methods used for the Farms Under Threat: State of the States project, version 2.0. Final Technical Report. Truckee, CA.

Cullen, J. A., C. L. Poli, R. J. Fletcher Jr., and D. Valle. 2022. Identifying latent behavioural states in animal movement with M4, a nonparametric Bayesian method. Methods in Ecology and Evolution 13:432–446.

Dancose, K., D. Fortin, and X. Guo. 2011. Mechanisms of functional connectivity: the case of free-ranging bison in a forest landscape. Ecological Applications 21:1871–1885.

Dewitz, J. and U.S. Geological Survey. 2021. National Land Cover Database (NLCD) 2019 Products (ver. 2.0, June 2021): U.S. Geological Survey data release. https://doi.org/10.5066/P9KZCM54.

Dickson, B. G., and P. Beier. 2007. Quantifying the influence of topographic position on cougar (Puma concolor) movement in southern California, USA. Journal of Zoology 271:270–277.

Elliot, N. B., S. A. Cushman, D. W. Macdonald, and A. J. Loveridge. 2014. The devil is in the dispersers: predictions of landscape connectivity change with demography. Journal of Applied Ecology 51:1169–1178.

Fieberg, J., J. Signer, B. Smith, and T. Avgar. 2021. A 'How to' guide for interpreting parameters in habitat-selection analyses. Journal of Animal Ecology 90:1027–1043.

Fortin, D., M.-E. Fortin, H. L. Beyer, T. Duchesne, S. Courant, and K. Dancose. 2009. Group-size-mediated habitat selection and group fusion–fission dynamics of bison under predation risk. Ecology 90:2480–2490.

Gorelick, N., M. Hancher, M. Dixon, S. Ilyushchenko, D. Thau, and R. Moore. 2017. Google Earth Engine: Planetary-scale geospatial analysis for everyone. Remote Sensing of Environment 202:18–27.

Hansen, M. C., P. V. Potapov, R. Moore, M. Hancher, S. A. Turubanova, A. Tyukavina, D. Thau, S. V. Stehman, S. J. Goetz, T. R. Loveland, A. Kommareddy, A. Egorov, L. Chini, C. O. Justice, and J. R. G. Townshend. 2013a. High-Resolution Global Maps of 21st-Century Forest Cover Change. Science 342:850–53.

Hansen, M. C., P. V. Potapov, R. Moore, M. Hancher, S. A. Turubanova, A. Tyukavina, D. Thau, S. V. Stehman, S. J. Goetz, T. R. Loveland, A. Kommareddy, A. Egorov, L. Chini, C. O. Justice, and J. R. G. Townshend. 2013b. High-Resolution Global Maps of 21st-Century Forest Cover Change. Science

342:850–853.
McIntosh, A. C. S., A. N. Gary, and S. L. Garman. 2009. Canopy structure on forest lands in western Oregon: differences among forest types and stand ages. Page 35. General Technical Report, Department of Agriculture, Forest Service, Pacific Northwest Research Station, Portland, OR.
Nickel, B. A., J. P. Suraci, A. C. Nisi, and C. C. Wilmers. 2021. Energetics and fear of humans constrain the spatial ecology of pumas. Proceedings of the National Academy of Sciences 118.
Nisi, A. C., J. P. Suraci, N. Ranc, L. G. Frank, A. Oriol-Cotterill, S. Ekwanga, T. M. Williams, and C. C. Wilmers. 2022. Temporal scale of habitat selection for large carnivores: Balancing energetics, risk and finding prey. Journal of Animal Ecology 91:182–195.
Sappington, J. M., K. M. Longshore, and D. B. Thompson. 2007. Quantifying Landscape Ruggedness for Animal Habitat Analysis: A Case Study Using Bighorn Sheep in the Mojave Desert. The Journal of Wildlife Management 71:1419–1426.
Shennan-Farpón, Y., P. Visconti, and K. Norris. 2021. Detecting ecological thresholds for biodiversity in tropical forests: Knowledge gaps and future directions. Biotropica 53:1276–1289.
Signer, J., J. Fieberg, and T. Avgar. 2017. Estimating utilization distributions from fitted step-selection functions. Ecosphere 8:e01771.
Tadono, T., H. Ishida, F. Oda, S. Naito, K. Minakawa, and H. Iwamoto. 2014. Precise Global DEM Generation by ALOS PRISM. ISPRS Annals of the Photogrammetry, Remote Sensing and Spatial Information Sciences II–4:71–76.
U.S. Environmental Protection Agency. 2021. Riparian Zones 2019.
U.S. Forest Service. 2016. Timber Harvests. Vector Digital Data.
Washington State Department of Natural Resources. 2017. Forest Practices Applications. Feature Layer.
Washington State Department of Transportation. 2023. Functional Class Data for State Routes. Feature Layer.
